# Direct inhibition of ACTN4 by ellagic acid limits breast cancer metastasis via regulation of β-catenin stabilization in cancer stem cells

**DOI:** 10.1186/s13046-017-0635-9

**Published:** 2017-12-02

**Authors:** Neng Wang, Qi Wang, Hailin Tang, Fengxue Zhang, Yifeng Zheng, Shengqi Wang, Jin Zhang, Zhiyu Wang, Xiaoming Xie

**Affiliations:** 1 Department of Breast Oncology, Sun Yat-sen University Cancer Center; State Key Laboratory of Oncology in South China; Collaborative Innovation Center of Cancer Medicine, Guangzhou, People’s Republic of China; 2 Guangdong Provincial Hospital of Chinese Medicine, The Second Clinical Medical College & The Research Center of Integrative Medicine, Guangzhou University of Chinese Medicine, Guangzhou Shi, China

**Keywords:** Cancer stem cells, Metastasis, ACTN4, Ellagic acid, β-catenin

## Abstract

**Background:**

Pharmacology-based target identification has become a novel strategy leading to the discovery of novel pathological biomarkers. Ellagic acid (EA), a dietary polyphenol compound, exhibits potent anticancer activities; however, the underlying mechanisms remain unclear. The current study sought to determine the role and regulation of ACTN4 expression in human breast cancer metastasis and EA-based therapy.

**Methods:**

The anti-metastasis ability of EA was validated by MMTV-PyMT mice and in vitro cell models. Drug affinity responsive target stability (DARTS) was utilized to identify ACTN4 as the direct target of EA. The metastatic regulated function of ACTN4 were assessed by cancer stem cells (CSCs)-related assays, including mammosphere formation, tumorigenic ability, reattachment differentiation, and signaling pathway analysis. The mechanisms of ACTN4 on β-catenin stabilization were investigated by western blotting, co-immunoprecipitation and ubiquitination assays. The clinical significance of ACTN4 was based on human tissue microarray (TMA) analysis and The Cancer Genome Atlas (TCGA) database exploration.

**Results:**

EA inhibited breast cancer growth and metastasis via directly targeting ACTN4 in vitro and in vivo, and was accompanied by a limited CSC population. ACTN4 knockdown resulted in the blockage of malignant cell proliferation, colony formation, and ameliorated metastasis potency. ACTN4-positive CSCs exhibited a higher ESA^+^ proportion, increased mammosphere-formation ability, and enhanced in vivo tumorigenesis ability. Mechanism exploration revealed that interruption of ACTN4/β-catenin interaction will result in the activation of β-catenin proteasome degradation. Increased ACTN4 expression was directly associated with the advanced cancer stage, an increased incidence of metastasis, and poor overall survival period.

**Conclusions:**

Taken together, our results suggest that ACTN4 plays an important role in breast CSCs-related metastasis and is a novel therapeutic target of EA treatment.

**Electronic supplementary material:**

The online version of this article (10.1186/s13046-017-0635-9) contains supplementary material, which is available to authorized users.

## Background

Although remarkable advances have been made in the evolution of therapeutic strategies and molecular elucidation of breast malignancies, recurrence and distant metastasis are still remained hard to cure and poorly understood [1, 2]. In 2016, it was reported that there will be 249,260 new breast cancer cases, of which 40,890 cases will be lethal to the U.S. female population [3]. Metastasis accounts for over 90% of the cases of mortality in cancer patients [4], and women with distant metastatic lesions have a significantly decreased 5-year survival rate compared with women with localized breast tumors (26.3% versus 98.8%) [5]. Strikingly, only 6% of the patients were initially diagnosed with metastatic breast cancer, and 20% to 50% of primary breast cancer patients would eventually develop invasive phenotypes [2]. Although overwhelming experimental evidence suggests that metastasis usually occurs at the advanced stages of cancer, some studies implied that tumor cells could be detected in the circulatory system as early as breast cancer initiation [6, 7]. However, the phenotype of circulating tumor cells is not necessarily uniform, only a minority of cancer cells could migrate to the distant organs and finally form the metastatic lesions [8, 9]. Hence, the identification of the metastatic cells that are capable of forming metastatic nodules and seeking targeted therapies to eliminate early metastasis are urgently needed in clinical management.

The discovery of CSCs sheds novel light in limiting metastasis. The CSC hypothesis supports the belief that cancer is largely driven by a subpopulation of malignant cells with the distinct ability of self-renewal, heterogeneous population generation, and tumor initiation [10]. Further, mounting studies suggest that CSCs exhibit high metastatic potential. Weng D et al. revealed that early metastatic cells that disseminated in the lungs displayed stem cell markers in the MMTV-PyMT mice model [9]. Liu et al. compared the gene expression differences between breast CSCs and normal mammary epithelial cells. There were 186 “invasive” genes that were identified, and they revealed a tight correlation to the overall survival and metastasis-free survival in breast cancer and other malignancies, including lung carcinoma, medulloblastoma, and prostate cancer [11]. Balic M et al. identified the existence of the stem-like cells within the bone marrow of breast cancer patients in the early phase [12]. All of these findings suggest that CSCs might be the precursors guiding cancer metastasis, and identification of key targets mediating CSCs metastasis is of great significance for improving cancer prognosis.

Target identification with natural phytochemicals has become an important strategy leading to the novel discovery of pathological biomarkers nowadays. Although there still is not a specific CSCs-targeting drug in the clinic at present, a lot of naturally occurring compounds have exhibited promising CSCs-limiting effects. Meanwhile, dietary supplements are specifically favored due to their wide safety profile, focus on multi-targets, and economic applications [13]. EA is one of the representative dietary polyphenols widely found in fruits and vegetables. Our previous study indicated that EA had significant suppressive effects on breast cancer growth and neoangiogenesis [14]. Meanwhile, extensive studies also proved EA to be an anti-cancer compound with multiple functions such as antioxidant, apoptosis induction, carcinogen-DNA binding blockage, and metastasis inhibition [15, 16]. However, little investigation has been carried out to explore its CSCs-limiting effects and the precise molecular target involved.

In the current study, we aimed to determine the direct molecular target of EA and its potential regulation effects on CSCs metastasis. DARTS analysis identified ACTN4 as the direct target of EA, and ACTN4 was found to closely correlate with CSCs’ self-renewal and metastatic abilities, poor survival period, and basal-like phenotype. Mechanism exploration revealed that interruption of ACTN4/β-catenin interaction will result in the activation of β-catenin proteasome degradation. Our findings would not only facilitate present understanding on the critical role of ACTN4 in breast CSCs metastasis, but also benefit for designing potentially more effective therapeutic strategies against breast cancer.

## Methods

### Chemicals and reagents

EA (over 97% purity) was provided by Alpha Aesar Company (Alfa Aesar, WardHill, MA). Dimethylsulfoxide (DMSO) was used to prepare the stock solution of EA and kept at −20 °C. 3-(4, 5-dimethylthiazol −2-yl) -2, 5-diphenyltetrazolium bromide (MTT), bovine serum albumin (BSA), insulin, 4′,6-diamidino-2-phenylindole (DAPI),

lithium chloride (LiCl), Akt inhibitor LY294002, cycloheximide (CHX) and proteasome inhibitor MG132, and were all purchased from Sigma (St. Louis, MO, USA).

### Cell culture

The human breast cancer cell lines MCF-7, BT-549, MDA-MB-231, SKBr3, MDA-MB-453, T47D, BT-474, HCC1937, and MCF-10A were purchased from the American Type Culture Collection (Manassas, VA, USA). The cells were cultured in medium (L15 for MDA-MB-231; RPMI-1640 for BT-549 and MCF-7; DMEM for SKBr3, MDA-MB-453, T47D, BT474, and HCC1937; and F12 for MCF-10A) at 37 °C in a humidified incubator with or without 5% CO2. 10% FBS and 1% penicillin and streptomycin (Gibco Life Technologies, Lofer, Austria) were added as supplements. The CSCs were sorted from MDA-MB-231 and BT-549 cells and maintained in DMEM/F12 medium. B27 (Invitrogen, Carlsbad, CA, USA), 5 μg/ml of insulin, 20 ng/ml of hEGF (BD Bioscience, Bedford, MA, USA), 1% penicillin/streptomycin and 0.4% BSA were added to DMEM/F12 medium for in vitro propagation.

### Breast cancer mice models and EA intervention

All animal procedures were performed in accordance with the institutional guidelines for the care and use of laboratory animals approved by the Animal Care and Use Committee of Guangdong Provincial Hospital of Chinese Medicine (the Ethics Approval Number 2015008) and the National Institutes of Health guide for the care and use of Laboratory animals. For MMTV-PyMT transgenic mice, 3-week-old female mice were randomly divided into the vehicle and the EA treatment groups, and EA (50 mg/kg/d) was given by intragastric administration from the Weeks 3–18 (*n* = 10 mice, total of 100 glands). For xenograft models, 10^6^ cells of MDA-MB-231 or BT-549 with or without genetic alternations were subcutaneously implanted into the mammary fat pads of female NOD/SCID mice at 4-week-old. After the tumor size reached over ~5 × 5 mm, EA (50 mg/kg/d) was given by intragastric administration for an additional 4 weeks. We monitored tumor formation and tumor size twice a week, and removed the tumors at the end-point of experiments for additional studies.

### India ink assay

Pulmonary metastatic lesions were imaged and calculated by India ink staining (15% India ink, 85% water, 3 drops of NH_4_OH/100 ml) through intra-tracheal injection. Feket’s solution (300 ml of 70% EtOH, 30 ml of 37% formaldehyde, and 5 ml of glacial acetic acid) were then prepared and washed the ink-stained lungs overnight to develop the white tumor nodules against a blue lung background.

### Immunohistochemistry and Immunofluorescence analysis

Tumor samples were paraffin-embedded and cut into 4 μm sections, and subsequently mounted onto poly-L-lysine-coated slides for immunohistochemistry analysis. The sections were then treated with xylene twice for 10 min and rehydrated with ethanol from 100% to 70% gradually and finally immersed in distilled water. H&E staining was applied to identify the lung metastatic lesions. For immunohistochemistry analysis, the sections were firstly treated with methanol (with 0.3% hydrogen peroxide) for 30 min to inactivate the endogenous peroxide at room temperature. Antigen retrieval was performed by heating the slides in sodium-citrate buffer, followed by permeabilization with 0.2% Triton X-100 for 15 min. After blocking with 10% goat serum, the sections were incubated with indicated primary antibody ACTN4 (Abcam, Cambridge, USA) at 4 °C overnight. DAB detection system (Dako A/S, Glostrup, Denmark) was applied as chromogenic agents according to the manufacturer’s instructions. Finally, sections were counterstained using Mayer’s hematoxylin, dehydrated, cleared, and mounted before examination. With regard to cellular immunofluorescence detection, 4% paraformaldehyde and 0.2% triton X-100 were firstly administrated to cells for 10 min. Following goat serum blocking for 60 min, the samples were co-incubated with primary antibodies including ACTN4 (Abcam, Cambridge, USA) and β-catenin (Cell Signaling Technology, Beverly, MA, USA) at 4 °C overnight, and subsequently labeled with fluorescence-conjugated secondary antibodies for 2 h at room temperature. 4′,6-diamidino-2-phenylindole (DAPI) was finally applied for nuclear staining and the signals were detected with NIKON TS2R fluorescence microscopy.

### Flow cytometry analysis

Single cell suspension were prepared and suspended in wash buffer (PBS containing 1% FBS) at a density of 10^7^/ml. Aldehyde dehydrogenase-based cell detection kit (Stem Cell Technologies, Grenoble, France) was used to detect the subpopulation of CSCs. Briefly, Aldehyde dehydrogenase (ALDH) substrate (Bodipy-aminoacetaldehyde) was co-incubated with 10^6^ cells for 45 min at 37 °C. Diethylaminobenzaldehyde (DEAB), an ALDH1 enzyme inhibitor, was added as a negative control. ALDH fluorescence was detected by a 488-nm blue laser. For CD44^+^/CD24^−^ detection or sorting assay, the cells were incubated with primary antibodies CD44-FITC, CD24-PE, and/or ACTN4-APC (BD Biosciences, San Diego, CA, USA) at 4 °C for 40 min. After washing with PBS, the cells were re-suspended and analyzed with FACSAria SORP (BD Biosciences, San Jose, CA, USA). FlowJo software was applied for data analysis.

### Cell proliferation and colony formation assays

MTT assay was conducted according to the manufacturer’s instructions. EA-treated or gene-modified cells were also seeded into 6-well plates at a density of 1000 cells/well to form colonies. After 2 weeks, the colonies were stained with Coomassie Blue and counted.

### Wound healing and transwell migration assays

For wound healing assay, the wound gap was recorded at 0, 24, or 48 h after the scratch made with a 10-μl pipette tip. Transwell assay was examined using 8-mm pore Transwell chambers (Milipore, Billerica, MA, USA).

### Mammosphere formation and tumorigenic evaluation assays

The sorted breast CSCs were subjected into mammosphere formation assay at a density of 1000 cells/well in ultra-low attachment plates. For tumorigenic assay, a series dilution of CSCs (10^5^, 10^4^, 10^3^) were injected into the mammary pads of NOD/SCID mice to compare the tumorigenic ratio and tumor initiating cell frequency calculated by Extreme Limiting Dilution Analysis (ELDA) software.

### Western blotting and co-immunoprecipitation analysis

Total protein extraction and western blot analysis were performed as described previously [17]. Antibodies for western blotting included ACTN4 (Abcam, Cambridge, USA), vimentin, E-cadherin (Abclonal, Cambridge, MA, USA), β-catenin, *p*-β-catenin (Ser33/37/Thr41), Akt, *p*-Akt (Ser473), GSK3β, *p*-GSK3β (Ser9), lamin B, β-actin (Cell Signaling Technology, MA, USA) as well as secondary anti-rabbit or anti-mouse antibodies. The interaction between ACTN4 and β-catenin were analyzed by co-immunoprecipitation analysis according to the instruction provided by Dynabeads Protein G immunoprecipitation kit (Invitrogen, Carlsbad, CA, USA).

### Darts

DARTS strategy was applied to identify the precise protein target of EA according to the protocol provided by *Lomenick* et al. [18]. Protein lysates from CSCs were quantified using bicinchoninic acid protein assay. Different concentrations of EA were then co-incubated with the lysates for 60 min at room temperature. The drug-protein interaction system was then treated with pronase (1:50, Roche Applied Science) to undergo proteolysis for 30 min at 4 °C. The lysates were finally denatured and subjected to western blotting analysis. The protective band was visualized by Coomassie bule staining and identified by MALDI-TOF-MS.

### In vitro ubiquitination assay

Ubiquitination detection assay was carried out according to the instruction provided by Ubiquitination Kit (UW9920, BioMol). The prepared cell lysates were firstly incubated with 100 nM E1, 2.5 mM UbcH5a as E2 [19], 20 U/ml of inorganic pyrophophatase (Sigma-Aldrich), 5 mM dithiothreitol, 5 mM Mg-ATP and 2.5 mM biotin-labelled ubiquitin in a 50 ml reaction system at 37 °C. After 4 h incubation, 50 ml of 2 × non-reducing gel-loading buffer was added to quench the reaction and subjected to SDS-PAGE analysis. After the proteins smaller than 70 kDa ran out, the gel was transferred onto PVDF membrane and immunoblotted with β-catenin antibody.

### Real-time PCR

TRIzol reagent (Invitrogen, Carlsbad, CA, USA) was applied to extract the total RNA, followed by reverse transcription reaction using the first-strand cDNA synthesis kit (Roche, Mannheim, Germany). SYBR Green kit (Roche, Mannheim, Germany) was utilized to perform real-time PCR analysis on Roche LightCycler 480 detector. PCR reaction condition was set as 95 °C for 10 min followed by 40 cycles of 95 °C for 10 s, 55 °C for 30 s, and 72 °C for 1 min. The target gene expression was calculated by 2^-△△Ct^ and normalized to the housekeeping gene control. The primers’ sequences were listed in Additional file 1: Table S1.

### Plasmids and siRNA construction and transfection

The pENTER vector plasmid carrying *ACTN4-*cDNA cloning and shACTN4 were purchased from Vigene Biosciences (Jinan, China) and transfected into cells using LipoFiter™ reagent (Hanbio Biotechnology Co, LTD. Shanghai, China). Scrambled plasmids were set as negative control. ACTN4 siRNA and their scrambled ones were bought from Invitrogen (Carlsbad, CA, USA) and transfected by X-tremeGENE siRNA transfection reagent (Roche Diagnostics, IN). The target protein expression was confirmed by western blotting. For the TCF/LEF luciferase assay, the TOPFLASH or FOPFLASH plasmids (Promega, Madison, WI) were transiently transfected into cells with or without EA treatment. pRL-TK plasmid was co-transfected for normalizing the transfection efficiency.

### RNA sequencing

RNA preparation, library construction and sequencing on BGISEQ-500 platform was performed at Beijing Genomics Institution (www.genomics.org.cn, BGI, Shenzhen, China). Genes expression levels were quantified by a software package called RSEM [20]. NOISeq method was used to screen differentially expressed genes (DEGs) between groups. Gene Ontology (GO) and pathway annotation and enrichment analyses were based on the Gene Ontology Database (http://www.geneontology.org /) and KEGG pathway database (http://www.genome.jp/kegg/), respectively. Statistical analysis was performed and DEGs were selected with the criteria of fold change ≥ 1.2, *P* ≤ 0.05.

### TMA and TCGA analysis

The tissue microarray HBre-Duc060CS-03 is commercially bought from Shanghai Outdo Biotech Company (http://www.superchip.com.cn/technology/Default.aspx, Shanghai, China) with National Human Genetic Resources Sharing Service Platform (Grant NO.: YCZYPT [2017] 02). All of the procedures were followed with the aforementioned immunohistochemistry assay as well as the manufacturer’s instructions. TCGA invasive breast carcinoma cancer study based on Agilent microarrays (TCGA, 2015) was applied to analyze *ACTN4* mRNA expression changes by the software cBioPortal (http://www.cbioportal.org). All the data retrieved from TCGA was supported by the guidelines built by TCGA Ethics, Law and Policy group, which are in compliance with the Helsinki Declaration (http://www.wma.net.u.vtrus.net/en/ 30publications/10policies/b3/index.html).

### Statistical analysis

Data analysis was performed with SPSS 13.0 software. The data were expressed as mean ± SD. Student’s *t*-test was used to compare the statistical difference between groups. The significance of multiple groups was compared using the one-way analysis of variance (ANOVA) followed by the Dunnett’s post hoc test. A value of *P* < 0.05 was considered significant.

## Results

### EA retards tumor growth and pulmonary metastasis

**Fig. 1:**
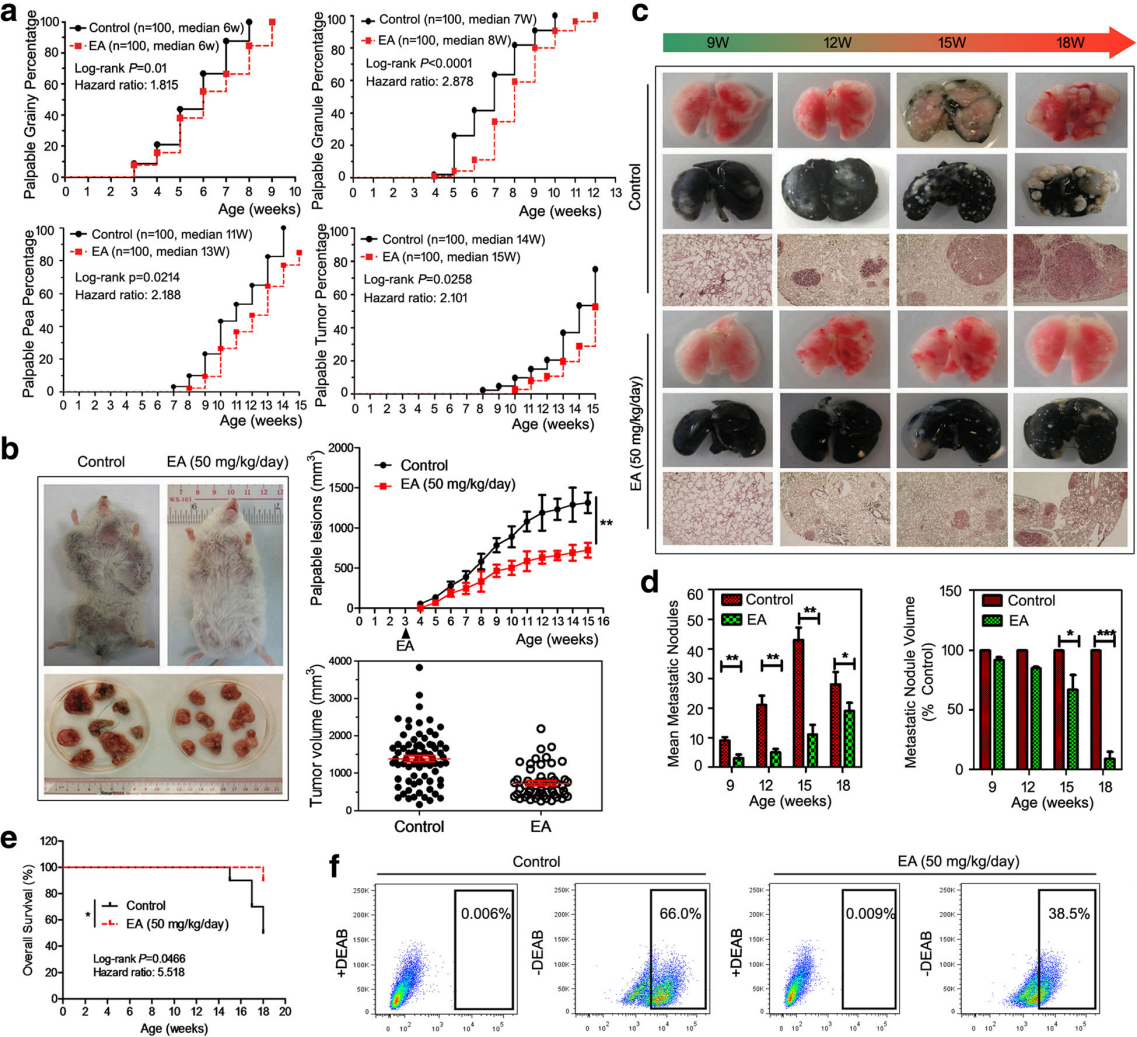
EA retards tumor growth and pulmonary metastasis. **a** Tumor incidence ratios between the vehicle and EA-treated groups in the MMTV-PyMT mice were compared using a log-rank test, highlighting the median time and the hazard ratio in each curve for grainy, granules, pea, or tumor phenotypes, respectively (*n* = 10 mice, total of 100 glands); **b** Representative images of tumors dissected from the vehicle or the EA-treated mice by 15 weeks (*left*); The curve showed EA resulted in significant tumor growth retardation compared with control at mice ages ranging from 3th to 15th weeks (*Upper right*); Tumor volume in the vehicle group (1383.53 ± 81.10 mm^3^) showed a significant difference compared with the EA-treated group (731.53 ± 59.32 mm^3^) (*Lower right*); **c** Representative images of lungs dissected from the vehicle or the EA-treated mice from the 9th to 18th weeks of mice age. India ink staining was used to identify pulmonary metastatic nodules of each group. H&E staining was utilized to observe tumor micromorphology of each group with 40-fold magnification; **d** The number and volume of metastatic nodules were measured from the 9th to 18th weeks of mice age. The results indicated that EA significantly inhibited the number and volume of metastatic nodules; **e** Mice overall survival (OS, %) in the control and EA treatment groups is shown by the Kaplan-Meier curve; **f** The ALDEFLUOR assay was to identify the population of CSCs in the metastatic nodules of the vehicle or the EA-treated MMTV-PyMT mice. ALDH^hi^ cells were significantly reduced in the EA-treated group (*P < 0.05, **P < 0.01, ***P < 0.001, values represented as the Mean ± SD, *n* = 10)

We initially aimed to characterize the anti-cancer activities of EA on the model of MMTV-PyMT transgenic mice, which produces spontaneous breast cancer. This mouse model is internationally well-accepted for preclinical breast cancer study based on its similar pathogenesis and characteristics with human breast malignancies. Here, descriptors (grainy, granules, pea, or tumor) are used to define the palpable phenotype for all mice glands each week [21, 22]. In particular, atypical hyperplasia is expected to be present in the 4-week-old mice with fine grains in the mammary glands. Since EA is a dietary compound, we began to feed the female MMTV-PyMT mice with EA (50 mg/kg/d) as early as 3 weeks of age by oral gavage every day. As shown in Fig. 1a, stage-specific progressions were significantly delayed by EA treatment compared with the vehicle group. Grainy lesions were found in 100% of the glands in Week 8 (vehicle) and week 9 (EA), respectively. The median time to 50% granules was 7 weeks for the vehicle group, whereas the EA group developed 50% granules on Week 8. By the week 14, all mammary glands (*n* = 100) developed pea-sized tumors in the vehicle group, while more than 10 glands in the EA treatment group were identified as free of lesions. EA treatment also obviously delayed the onset of measurable tumors by approximately 2 weeks compared to the vehicle. In addition, mammary tumors in the vehicle group exhibited a significantly increased tumor volume with a more necrotic and hemorrhagic region, suggesting that EA resulted in significant tumor growth retardation (Fig. 1b). Cancer progression was also slowed down if the EA treatment was started after tumor growth in MMTV-PyMT mice at ages ranging from 6th to 15th weeks (Additional file 2: Figure. S1A).

Previous studies have indicated that the MMTV-PyMT mice tend to develop luminal-type mammary adenomas with a tendency of lung metastasis lesions on week 10–15 [21, 22]. Therefore, we prolonged the administration periods of EA for an additional 3 weeks to examine its inhibition effects on pulmonary metastasis. Visual differences in the lung specimens were observed from Weeks 9–18 between the 2 groups. India ink staining and hematoxylin and eosin (H&E) staining further confirmed the suppressive effects of EA on the pulmonary metastasis, especially at the end-stage progression (Fig. 1c). In particular, EA administration obviously inhibited the number and volume of metastatic nodules. The mean metastatic nodules in the EA intervention group on Week 15 was significantly decreased to over 50% compared with the vehicle group, while EA elicited a dramatic 90% reduction in the nodule volume compared to the vehicle groups by Week 18 **(**Fig. 1d **)**. We additionally investigated the overall survival (OS) of mice with or without EA (50 mg/kg/day) at the end of treatment on week 18. The death ratio is 5/10 and 1/10 in control and EA group, respectively. The Kaplan-Meier curve analysis demonstrated that EA significantly prolonged the survival time of MMTV-PyMT mice, possibly due to the decreased tumor burden and the limited lung metastasis (*P* = 0.0265, Fig. 1e). To thoroughly investigate the potent anti-metastasis effects of EA, we harvested metastatic nodules to detect the CSC population by the ALDH assay. Compared with the DEAB negative control, the CSCs in metastatic nodules of the vehicle group reached approximately 66%, while EA treatment dramatically ALDH^hi^ cells to 38.5% **(**Fig. 1f **)**. This information hinted that EA might antagonize breast cancer progression and subsequent pulmonary metastasis via suppressing breast CSC activity. Finally, little adverse events, including decreased appetite, weight loss, ruffling fur, or abnormal behavior, was observed in the mice throughout the whole experiment, suggesting that the anti-cancer bioactivities of EA might not be attributed to its toxicity effects (Additional file 2: Figure. S1B).

### EA inhibits breast cancer metastasis associating with CSC limitation

**Fig. 2:**
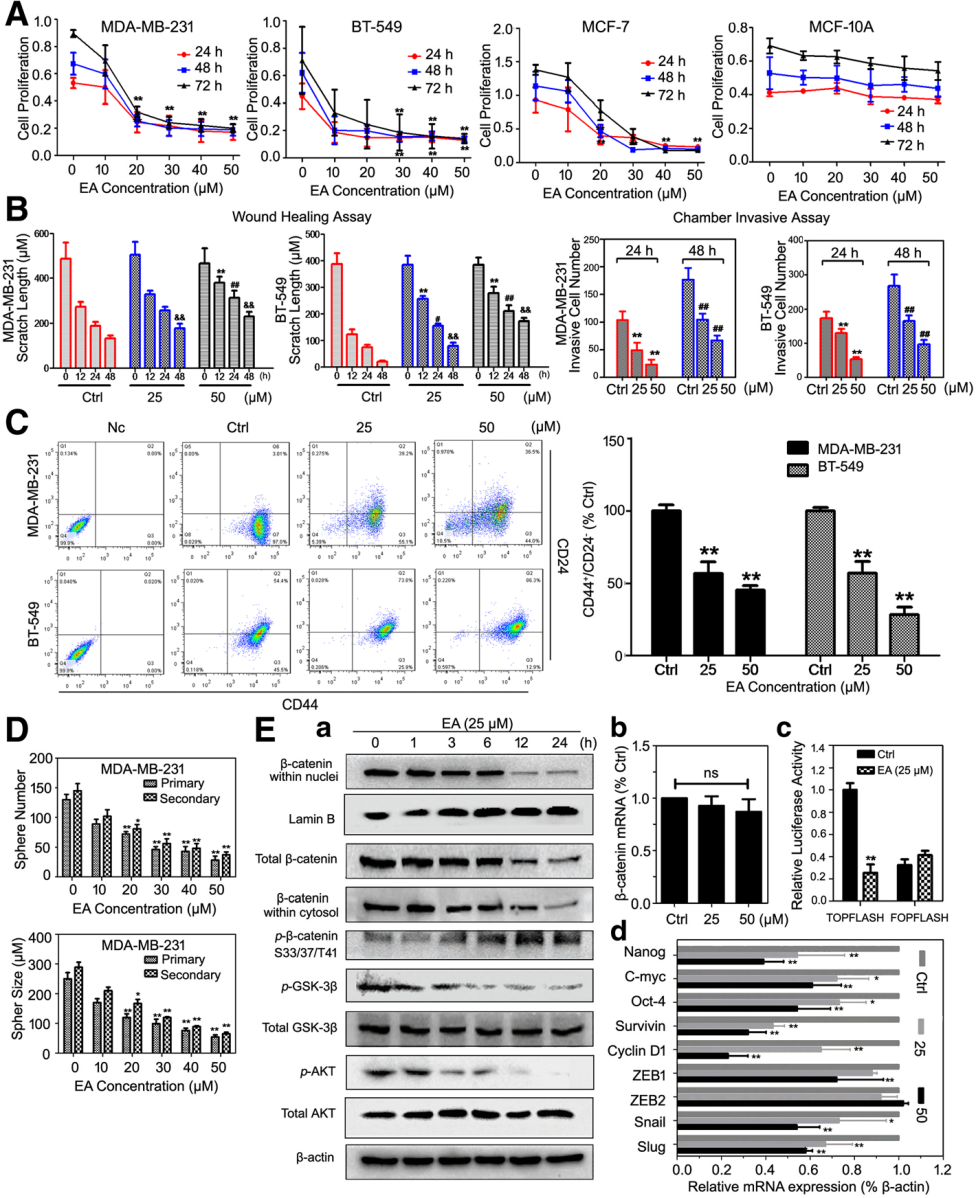
EA inhibits breast cancer cell proliferation, migration, and invasion abilities associated with CSC limitation. **(A)** EA suppressed the proliferation of the breast cancer cell lines MDA-MB-231, BT-549, and MCF-7, while posing little inhibitory effects on the human normal mammary epithelial cell MCF-10A (**P < 0.01 versus control, values represented as the mean ± SD, *n* = 3) at different dose- and time-intervals indicated; **(B)** The wound healing and chamber invasive assay revealed that breast cancer cell migration and invasion were inhibited by EA in a time- and dose-dependent manner (**P < 0.01 versus control at 12 h, ^#^ *P* < 0.05, ^##^ *P* < 0.01 versus control at 24 h, ^&&^ *P* < 0.01 versus control at 48 h, values represented as the mean ± SD, *n* = 3); **(C)** EA dose-dependently reduced the CSC populations in the breast cancer cells. Representative dot plots of CD44^+^CD24^−/low^ cell surface markers in the breast cancer cells MDA-MB-231 and BT-549 (**P < 0.01 versus control, values represented as the mean ± SD, *n* = 3); **(D)** EA limited the primary and secondary CSC mammosphere of the MDA-MB-231 and BT-549 cells in a dose-dependent manner (*P < 0.05, **P < 0.01, values represented as the mean SD, *n* = 3); **(E) a.** Western blotting analysis validated that EA elevated p-β-catenin, while inhibiting *p*-GSK-3β and *p*-AKT in the MDA-MB-231 stem-like cell lysates time-dependently; **b.** EA did not suppress the β-catenin mRNA level after 24 h administration; **c.** EA inhibited its transcriptional activity as detected by the TCF/LEF luciferase assay (**P < 0.01, values represented as the mean ± SD, *n* = 3); **d.** The relative mRNA expressions of the β-catenin downstream genes were inhibited by EA in the MDA-MB-231 stem-like cell lysates (*P < 0.05, **P < 0.01, values represented as the mean ± SD, *n* = 3)

After in vivo validation, we continued to validate whether EA could exert anti-proliferative effects on the representative breast cancer cell lines MDA-MB-231 (basal-like), BT-549 (basal-like), and MCF-7 (luminal-like) by MTT assay. As shown in Fig. 2A, EA could inhibit cell proliferation in a dose-dependent manner, with an IC50 of approximately 25 μM for MDA-MB-231, 20 μM for BT-549, and 30 μM for MCF7 within 24 h. EA administration continued to significantly suppress breast cancer cells over the next 72 h, whereas it did not significantly affect the proliferation of the normal mammary cells MCF-10A from 24 to 72 h. Furthermore, flow cytometry analysis indicated that EA increased the subpopulation of cells undergoing late apoptosis, and arrested the cell cycle in the breast cancer cells mainly at the S&G2/M phases (Additional file 3: Figure. S2). These data proved that EA elicited a cell growth–inhibitory effect on the breast cancer cell lines, particularly on basal-like phenotypes.

Because EA administration resulted in the decreased metastatic nodules, we next investigated the in vitro effects of EA on breast cancer cell migration and invasion. The influence of EA on the migrate ability of breast cancer cells was assessed by wound healing assay. The gap width in the control group was narrowed more rapidly than that of EA group from 0 to 48 h, indicating that EA could dose- and time-dependently inhibit the migration ability of both MDA-MB-231 and BT-549 cells. For the chamber invasion assay, the results showed that the number of invasive cells was significantly reduced following EA treatment, demonstrating that EA treatment profoundly impaired the invasion capacity of the cancer cells **(**Fig. 2B; Additional file 4: Figures. S3 and S4**)**. Since metastasis is closely associated with stemness properties, we then sought to determine if the anti-metastasis ability of EA could be due to its pharmacological targeting of CSCs. As expected, EA dose-dependently decreased the CD44^+^CD24^−/low^ phenotypes in both CSC-enriched MDA-MB-231 and BT549 cells after 24 h **(**Fig. 2C **)**. To further assess the influences of EA on the self-renewal ability of CSCs, we sorted CD44^+^/CD24^−/low^ subpopulations from MDA-MB-231 cells and conducted mammosphere formation assay. We exposed mammospheres to increasing concentrations of EA and then cultured them for 1 additional passage without EA administration. EA intervention was found to be associated with a decreased number and size of the stem-like cell spheres in MDA-MB-231cells. In particular, the decreased number and size of sphere-forming cells in the subsequent passages further demonstrated that the abrogated CSC self-renewal capacity elicited by EA could possibly be effective and heritable **(**Fig. 2D **)**.

β-catenin is one of the structural molecules mediating the cell-cell adhesion for cancer cell migration, and is also known for a mediator of cellular signaling pathways for CSC maintenance. Given its distinct role in the epithelial-mesenchymal transition (EMT) process and CSC properties, we tested the influences of EA on β-catenin expression and distribution in CSCs derived from MDA-MB-231 cells. Our results found that the cytosolic and nuclear β-catenin expressions were simultaneously downregulated by EA via the Akt/GSK-3β pathway **(**Fig. 2E(a)**)**, whereas it posed little effect on the β-catenin mRNA level after 24 h EA treatment **(**Fig. 2E(b)), indicating that the β-catenin suppression by the EA treatment might have possibly occurred at the posttranslational level rather than transcriptionally. A TCF/LEF luciferase reporter study also revealed that EA potently inhibited β-catenin transcription activity, and possibly resulting in the impaired expressions of the stem-like markers (Nangog, C-myc, Oct-4, and Survivin), as well as the cancer growth and metastasis-associated molecules (Cyclin D1, Snail, ZEB1, and Slug) (Fig. 2E(c) and 2E(d**))**. Collectively, EA inhibits breast cancer metastasis possibly by limiting CSCs.

### Identification of ACTN4 as the direct target of EA in breast CSCs

**Fig. 3:**
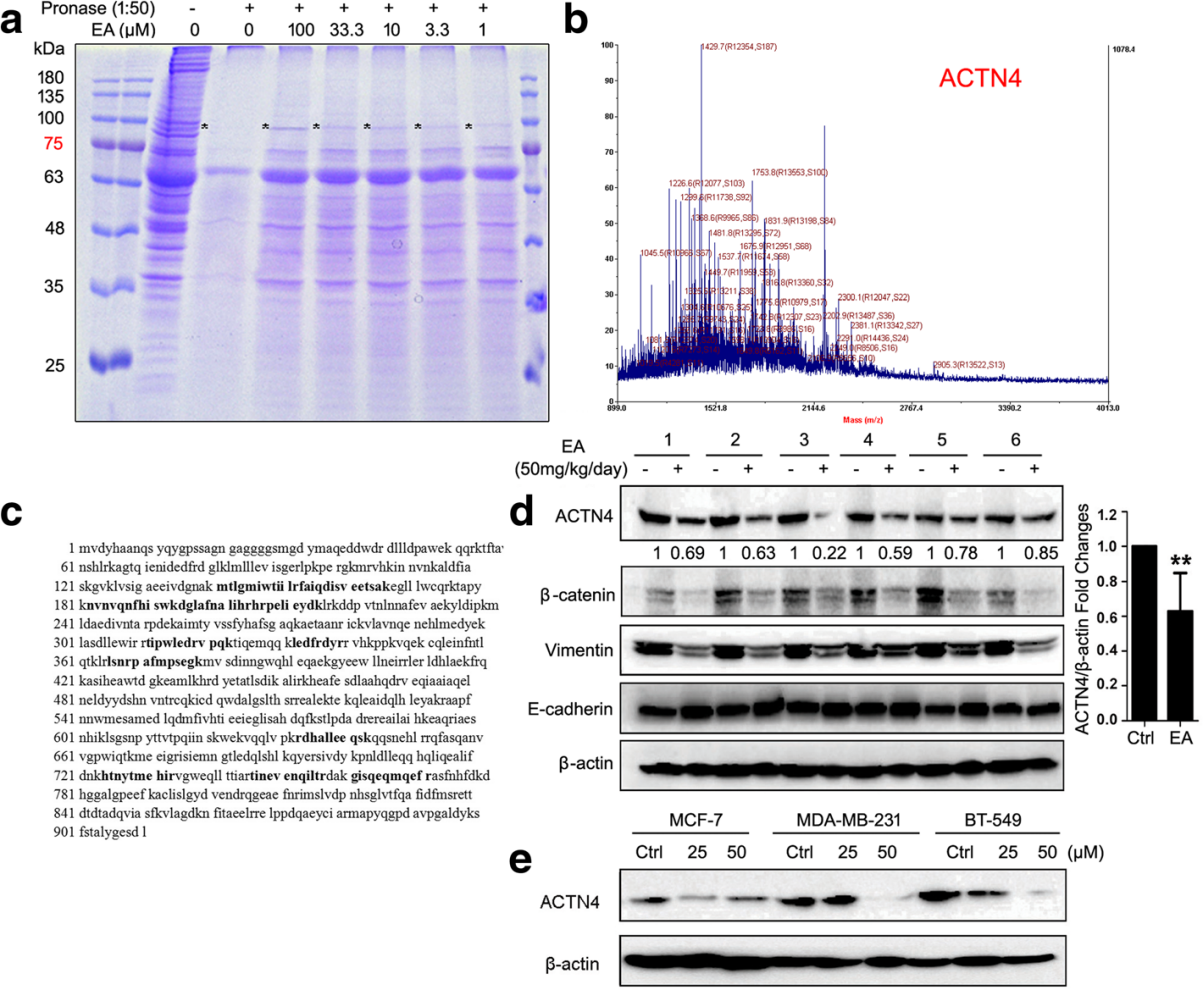
Identification of ACTN4 as the direct target of EA in breast CSCs. **a** MDA-MB-231 CSCs were treated with EA at varying concentrations, a 100-kDa protein band was protected from digestion in a dose-dependent manner. The arrowhead indicates the 100-kDa protein; **b** Mass spectrogram identified the 100-kDa-targeted protein of EA as ACTN4; **c** Amino acid sequence of actinin-4. *Bold letters*: peptides that correspond to peaks identified by mass spectrometry; **d** EA administration significantly led to an average suppression in the ACTN4 expression levels, accompanied by inhibited expressions of β-catenin and vimentin in six tumors from EA-treated MMTV-PyMT transgenic mice tumors. A quantitative measurement was conducted to analyze with Image J (**P < 0.01, values represented as the mean ± SD, *n* = 6). Cell lysates of Lanes 1, 3, 5, 7, 9 and 11 came from six MMTV-PyMT transgenic mice in vehicle groups, while proteins of Lanes 2, 4, 6, 8, 10 and 12 belonged to six mice in EA treatment groups; **e** EA inhibited ACTN4 expression in the MDA-MB-231, BT-549, and MCF-7 cells by western blot analysis

Although it was demonstrated that EA suppressed breast CSCs through inhibiting β-catenin signaling, the direct molecular target of EA was still remained unclear. The DARTS strategy is designed to identify the direct binding target of small molecules based on the lower susceptibility of chemical-protein complex to proteolysis [18, 23]. Following EA treatment, DARTS identified a protective band near 100 kDa presenting as a dose-dependent manner **(**Fig. 3a **)**. MALDI-TOF-MS subsequently identified the band as ACTN4 **(**Fig. 3b and c **)**. Immunoblotting analysis further revealed that EA treatment significantly led to an average suppression of ACTN4 expression in tumors from six pairs of MMTV-PyMT transgenic mice, accompanying with decreased expressions of vimentin and β-catenin **(**Fig. 3d **)**. The reduced expression of ACTN4 was further confirmed at protein levels in EA–treated breast cancer cell lines **(**Fig. 3e **)**. Taken together, our results showed that EA could suppress the ACTN4 expression both in vitro and in vivo, suggesting that ACTN4 might be a critical contributor to breast cancer metastasis.

**Fig. 4:**
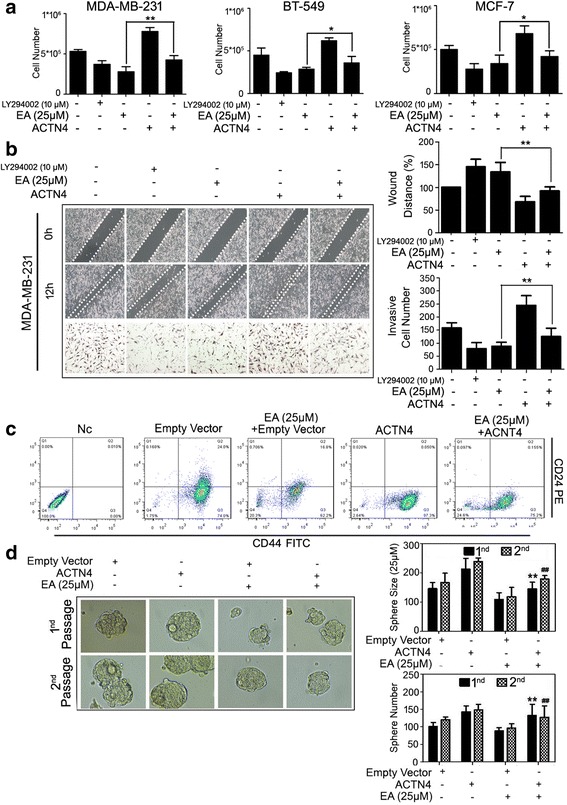
ACTN4 overexpression abrogated the anti-cancer and anti-metastatic effects induced by EA on breast cancer. **a** MDA-MB-231, BT-549 and MCF-7 cells were transfected with ACTN4 recombinant plasmids and treated with or without 25 μM EA for 24 h. LY294002 (10 μM) was used as a positive control for this assay. The findings revealed that ACTN4 overexpression enhanced breast cancer cell number, and effectively restored the inhibition effects of EA on breast cancer cells (*P < 0.05, **P < 0.01, values represented as the mean ± SD, *n* = 3); **b** ACTN4 overexpression endowed EA-treated cells with increased migratory and invasive ability (**P < 0.01, values represented as the mean ± SD, *n* = 3). LY294002 (10 μM) was used as a positive control for this assay; **c** ACTN4 overexpression abrogated the CSC suppressive effects induced by EA, presenting as increased CSC proportion and enhanced sphere formation capability (**P < 0.01 EA + empty vector group versus EA + ACTN4 group in 1st CSC sphere generation, ^##^ *P* < 0.01 EA + empty vector group versus EA + ACTN4 group in 2nd CSC sphere generation, values represented as the mean ± SD, n = 3)

To directly demonstrate whether ACTN4 is critical for EA action, we transfected ACTN4 recombinant plasmid in MDA-MB-231, BT-549 and MCF-7 cells with or without EA. According to previous studies, some known inhibitor particularly AKT inhibitor LY294002 could potently suppress breast cancer proliferation, migration and invasion [17, 24, 25], therefore we also included LY294002 (10 μM) as positive control in the following cell proliferation and migration assays. Proliferation analysis revealed that ACTN4 overexpression enhanced breast cancer cell number, and effectively restored the inhibition effects of EA on breast cancer cells **(**Fig. 4a **)**. In addition, ACTN4 overexpression endowed EA-treated cells with increased migratory and invasive ability, further implying the significant role of ACTN4 in mediating the metastasis inhibition effects of EA **(**Fig. 4b **)**. The results also gave us a comparison picture about EA and AKT inhibitor LY294002 on their anti-cancer potential. Meanwhile, compared with cells transfected with empty vector, ACTN4 overexpression not only promoted CSC properties but also abrogated the CSC suppressive effects induced by EA, presenting as increased CSC proportion and enhanced sphere formation capability **(**Fig. 4c **)**.

**Fig. 5:**
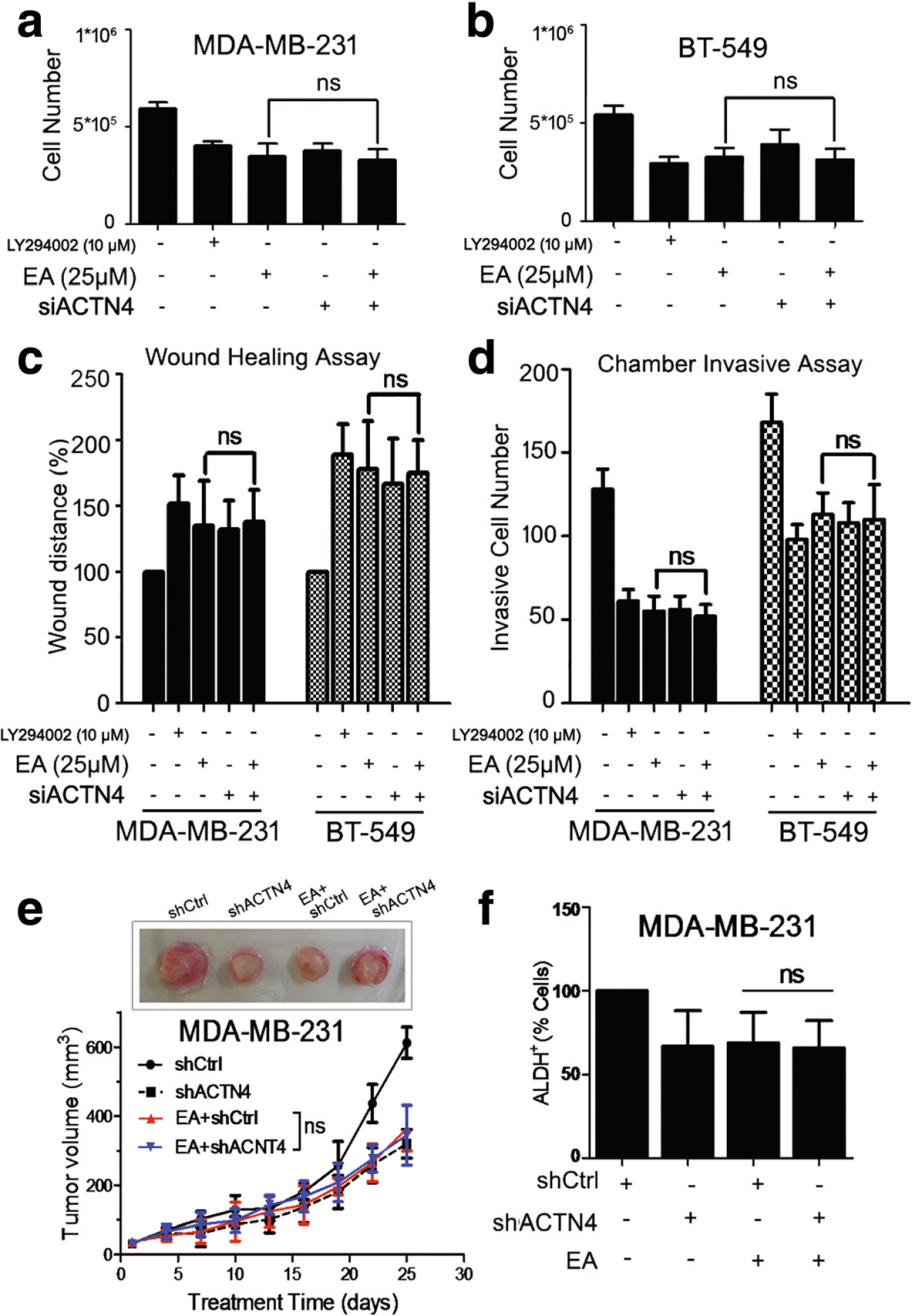
ACTN4 knockdown exerted no additive anti-cancer and anti-metastatic effects induced by EA on breast cancer. **a** MDA-MB-231 and (**b**) BT-549 cells were transfected with siACTN4 for 48 h and treated with or without 25 μM EA for additional 24 h. LY294002 (10 μM) was used as a positive control substance for this assay. The findings revealed that siACTN4 did not aggravate the inhibition effects of EA on breast cancer cells when used in combination; **c-d** siACTN4 did not significantly aggravate the inhibition effects of EA on cell migration and invasion of MDA-MB-231 cells. LY294002 (10 μM) was used as a positive control substance for this assay; **e** For xenograft models, 10^6^ cells of MDA-MB-231 with or without ACTN4 knockdown were subcutaneously implanted into the mammary fat pads of female NOD/SCID mice at 4-week-old in the presence or absence of EA (50 mg/kg/d). There was no additive inhibitory effects on tumor growth when combining EA with ACTN4 knockdown; **f** The ALDEFLUOR assay showed that the ALDH^+^ subpopulations in the tumor essence between EA alone and EA plus shACTN4 groups

Together, these data demonstrated the significant role of ACTN4 in mediating the metastasis inhibition effects of EA. The findings were further supported by the fact that siACTN4 did not significantly aggravate the inhibition effects of EA on cell proliferation **(**Fig. 5a and b **)** and migration **(**Fig. 5c and d **)** of breast cancer cells. For xenograft models, 10^6^ cells of MDA-MB-231 with or without ACTN4 knockdown were subcutaneously implanted into the mammary fat pads of female NOD/SCID mice at 4-week-old in the presence or absence of EA (50 mg/kg/d). There was no additive inhibitory effects on tumor growth when combining EA with ACTN4 knockdown **(**Fig. 5e **)**. Given that CSCs represent high metastatic potential [9, 10], we further detected the CSC population in the tumor essence by ALDH assay. The ALDH^+^ subpopulations in EA plus shACTN4 group showed no significant difference with those in EA group **(**Fig. 5f **)**. To thoroughly investigate the global effects of EA treatment and ACTN4 knockdown on breast cancer, we additionally perform a global RNA-seq for MDA-MB-231 cells treated with EA or shACTN4 using a BGISEQ-500 by the Beijing Genomic Institution (www.genomics.org.cn, BGI, Shenzhen, China). Subsequent analysis identified 510 differentially expressed genes (DEGs) in control *v.s.* EA-treated group as well as 399 DEGs in control *v.s.* ACTN4 knockdown group, respectively (fold change ≥ 1.2, *P* ≤ 0.05). The heat map columns and venn diagrams of altered DEGs were shown in Additional file 5: Figure. S5A and B. For Gene Ontology (GO) analysis (Additional file 5: Figure. S5C), the terms existing in both DEG groups encompassed various biological processes during cancer progression, such as localization/locomotion, cell death/proliferation, extracellular region/space, cytoskeleton as well as binding activity, etc. For Kyoto Encyclopedia of Genes and Genomes (KEGG) enrichment analysis (Additional file 5: Figure. S5D), the interfered pathways co-existing in both DEGs were mainly responsible for supporting cancer growth and invasion like focal adhesion signaling, signaling pathways regulating pluripotency of stem cells, Wnt signaling, PI3K-Akt signaling, etc. Of note, the results demonstrated that EA treatment and ACTN4 knockdown possibly changed the same group of target genes, suggesting that EA treatment and ACTIN4 knockdown might change the same group of target genes.

### ACTN4 promotes breast cancer growth and metastasis

**Fig. 6:**
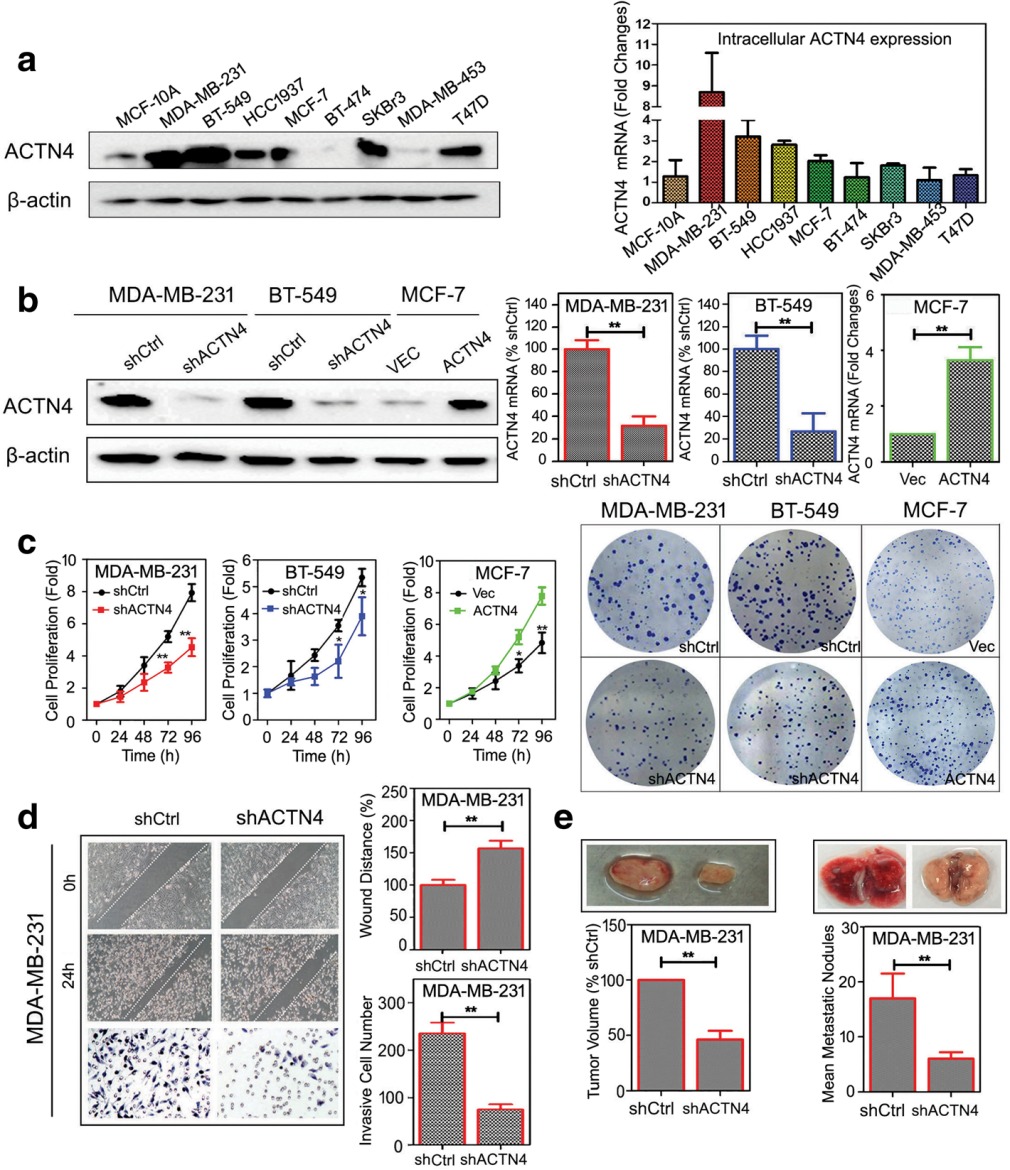
ACTN4 promotes breast cancer proliferation and metastasis in vitro and in vivo. **a** Intracellular expression of ACTN4 was determined by Western blot (left) and real-time PCR (right) analysis, respectively; **b** ACTN4 expression was modified by transfecting recombinant plasmid or its shRNA in breast cancer cells and subjected to Western blotting (left) and real-time PCR (right) validation; **c** MTT and colony formation assay showed that ACTN4 silencing abrogated breast cancer cell proliferation while its overexpression promoted cell growth; **d** ACTN4 silencing inhibited the migration and invasion abilities of MDA-MB-231 cells; **e** ACTN4 silencing inhibited breast cancer growth and lung metastasis in vivo (*P < 0.05, **P < 0.01 versus control, values represented as the mean ± SD, n = 3)

We next explored whether the anti-cancer and anti-metastasis effects of EA were ACTN4-dependant. Immunoblotting and real-time PCR analysis revealed that the enhanced expression of ACTN4 was observed in several basal-like breast cancer cell lines (MDA-MB-231, BT-549, and HCC1937) compared with the normal mammary cell line MCF-10A **(**Fig. 6a **)**. The stable ACTN4-knockdown cells (MDA-MB-231 shACTN4 and BT-549shACTN4) and ACTN4-overexpressing cells (MCF-7ACTN4) were established by shRNA transfection and plasmids. ACTN4 expression in the gene-modified cells was confirmed by western blot analysis and real-time PCR **(**Fig. 6b **)**. It was found that the ACTN4-knockdown cells maintained an epithelial cell appearance, whereas their shRNA control exhibited a spindle-like mesenchymal cell morphology. In addition, its absence led to a blockage of malignant cell proliferation, impaired colony formation, and ameliorated metastasis potency in vitro (Fig. 6c and d). Moreover, tumor formation in the nude mice demonstrated that ACTN4 knockdown could significantly inhibit tumor growth and lung metastasis (Fig. 6e).

### ACTN4 facilitates breast CSCs self-renewal by stabilizing β-catenin signaling

**Fig. 7:**
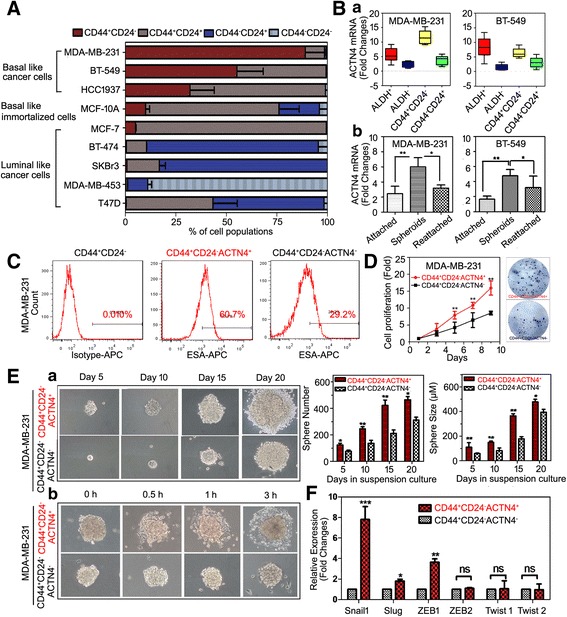
ACTN4 is closely correlated with the CSCs phenotype. **(A)** The expression panel of CD44 and CD24 in breast cancer cell lines; **(B) a.** ACTN4 expression was significantly elevated in the ALDEFLUOR-positive cells and CD44^+^/CD24^−^ phenotypes; **b.** ACTN4 expression was greatly enhanced in the undifferentiated CSC spheroids mammospheres (*P < 0.05, **P < 0.01 versus CSC spheres, values represented as the mean ± SD, n = 3); **(C)** EpCAM expression was significantly up-regulated in the ACTN4-positive CSCs by flow cytometry analysis; **(D)** CD44^+^/CD24^−^/ACTN4^+^ cells displayed enhanced proliferation in MDA-MB-231 cells (**P < 0.01, values represented as the mean ± SD, *n* = 3); **(E)** CD44^+^/CD24^−^/ACTN4^+^ cells represent a distinct population of CSCs with higher self-renewal **(c)** and adherence-promoting **(b)** capacity (*P < 0.05, **P < 0.01 versus CD44^+^/CD24^−^/ACTN4^−^ cells, values represented as the mean ± SD, *n* = 3); **(F)** The relative mRNA expressions of EMT markers in CD44^+^/CD24^−^/ACTN4^+^ cell lysates (*P < 0.05, **P < 0.01, values represented as the mean u SD, *n* = 3)

The aforementioned results prompted us to explore the correlation between ACTN4 and CSC phenotypes. Among 9 breast cancer cell lines examined, 3 cell lines (MDA-MB-231, BT-549, and HCC1937) contained a relatively higher percentage (>30%) of CD44^+^/CD24^−^ cells, indicating that the high percentage of CD44^+^/CD24^−^ cells directly associates with a basal phenotype **(**Figure 7A). Nevertheless, recent studies suggested that CD44^+^/CD24^−^ cells do not correlate with distant metastasis or with the clinical outcome [26]. Given that ACTN4 might be a bridge connecting CSCs and metastasis function in breast cancer, particularly for the basal-like populations, we then investigated the expression of ACTN4 in ALDEFLOUR-positive cells or CD44^+^/CD24^−^ populations. ALDEFLOUR-positive cells contained a higher ACTN4 expression compared with the ALDEFLOUR-negative cells. ACTN4 expression was also higher in the CD44^+^/CD24^−^ cells in comparison with the non-CD44^+^/CD24^−^ cells. CSC spheroids are differentiated after reseeding under adherent conditions **(**Fig. 7B(a)). We then detected the ACTN4 expressions in the non-CSCs cells in adherent culture conditions, undifferentiated CSC spheroids, and CSCs in adherence-promoting plates. As expected, the results revealed ACTN4 differences among the three groups following a pattern of CSC spheres > CSC differentiating forms > non-CSCs **(**Fig. 7B(b)). Taken together, these results showed an enrichment of ACTN4 in breast CSC phenotype.

To further extend a link between ACTN4 and CSC phenotype, we sorted ACTN4-positive (CD44^+^/CD24^−^/ACTN4^+^) and -negative cells (CD44^+^/CD24^−^/ACTN4^−^) from MDA-MB-231 CSCs and examined them for their stem-like functions. A previous study demonstrated that CD44^+^/CD24^−^/ESA^+^ was 50-fold enriched in the ability to form breast tumors relative to unfractionated tumor cells [27]. In Fig. 7C, FACS analyses demonstrated that the proportion of EpCAM^+^ cells in CD44^+^/CD24^−^/ACTN4^+^ populations tended to be larger than that in CD44^+^/CD24^−^/ACTN4^−^ pools, strongly suggesting that the pattern of ACTN4 expression in the CD44^+^/CD24^−^ phenotype may be similar to that of EpCAM, and that ACTN4 may play a significant role in the self-renewal capacity of CD44^+^/CD24^−^ cells. We next explored the growth potential of CD44^+^/CD24^−^/ACTN4^+^ and CD44^+^/CD24^−^/ACTN4^−^ cells. In cell proliferation assays, it was found that ACTN4^+^ CSC-like cells showed a significantly greater proliferation capability than its ACTN4^−^ counterparts **(**Figure 7D). The ACTN4^+^ cells formed larger and increased the number of mammospheres compared with the ACTN4^−^ cells (Fig. 7E(a)). Meanwhile, the ACTN4^+^ cells also had a greater ability to reattach than the ACTN4^−^ cells (Fig. 7E(b)), and influenced the transcription of EMT markers (Figure 7F). Thus, CD44^+^/CD24^−^/ACTN4^+^ represent a distinctive invasive phenotype with a higher self-renewal and adherence-promoting capacity of CSCs. Moreover, subcutaneous injection of ACTN4-expressing CD44^+^/CD24^−^ cells resulted in an increased incidence of tumor formation, even when only 1000 cells were implanted, further verifying the tumorigenic significance of ACTN4 on breast cancer **(**Additional file 1: Table S2**)**.

**Fig. 8:**
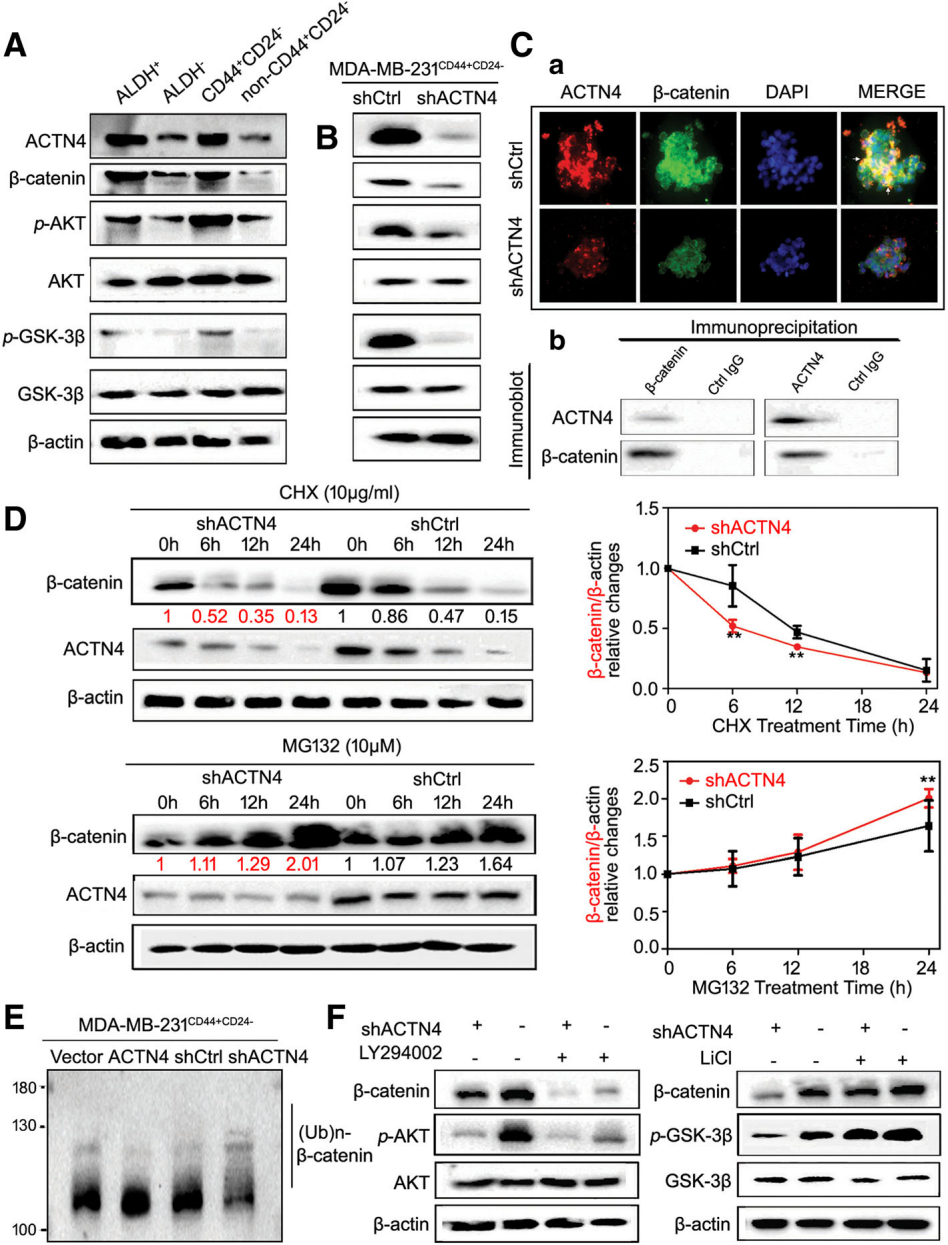
ACTN4 sustains CSCs properties by promoting β-catenin stabilization. **(A)** The expressions of ACTN4 and β-catenin pathway signaling were elevated in the sorted ALDEFLUOR-positive and CD44^+^/CD24^−^ cells; **(B)** ACTN4 knockdown in CSCs resulted in decreased β-catenin and p-AKT/GSK3β expressions; **(C) a.** the immunofluorescence assay showed the co-localization of ACTN4 and β-catenin was mainly located in the cytosol (arrowheads); **b.** the immunoprecipitation assay revealed the direct molecular interaction between β-catenin and ACTN4; **(D)** Breast CSCs were transfected with shACTN4, treated with CHX (10 μg/ml), and MG132 (10 μM) for the indicated time and immunoblotted, and the results showed that ACTN4 silencing could promote β-catenin proteasome degradation. A quantitative measurement was conducted to further analyze with Image J (**P < 0.01, values represented as the mean ± SD, n = 3). After CHX treatment, the half-life of β-catenin degradation in shCtrl cells was estimated to be approximately 12.1 h, whereas it was 4.5 h in ACTN4 silencing cells based on Engauge Digitizer software analysis; **(E)** ACTN4 overexpression caused a loss of poly-ubiquitination accumulation of β-catenin, while its knockdown was found to increase β-catenin ubiquitination and its consequent degradation, presenting as accumulation of ubiquitinated β-catenin; **(F)** Breast CSCs transfected with shACTN4 were treated with LY294002 (10 μM) and LiCl (10 μM) and immunoblotted, and the results indicated that ACTN4 silencing activated β-catenin proteasome degradation via modulating GSK3β/Akt phosphorylation

We continued to thoroughly investigate the underlying mechanisms of ACTN4 mediation of the breast CSC properties. ACTN4 expression was also found to be correlated positively with the Akt/GSK-3β/β-catenin levels (Figure 8A). Moreover, ACTN4 silencing led to a significant inhibition of β-catenin signaling and downregulation of AKT/GSK-3β phosphorylation in MDA-MB-231^CD44+/CD24-^ populations, which was consistent with the molecular mechanisms of EA (Figure 8B). Double immunofluorescence analysis revealed that β-catenin and ACTN4 were overlapped mainly in the cytosol of breast CSCs sorted from MDA-MB-231 (Fig. 8C(a), arrowheads). The immunoprecipitation assay further confirmed the direct interaction between β-catenin and actinin-4 (Fig. 8C(b)). Once the cytosolic interaction between ACTN4 and β-catenin was interrupted, β-catenin stabilization might be destroyed and subsequently result in the decreased nuclear localization. Since the proteasome degradation pathway is the primary posttranslational regulatory mechanism for decreasing the β-catenin level, we therefore investigated whether ACTN4 inactivation could interrupt β-catenin stabilization by activating its proteasome degradation pathway. We firstly examined the effect of the protein synthesis inhibitor CHX on β-catenin level in ACTN4-silenced cells. After CHX treatment, the half-life of β-catenin degradation in shCtrl cells was estimated to be approximately 12.1 h whereas it was 4.5 h in ACTN4 silencing cells, implying that β-catenin degradation was accelerated following ACTN4 knockdown. By contrast, β-catenin degradation induced by ACTN4 knockdown was blocked following the treatment of proteasome inhibitor MG132, further confirming that the proteasome degradation pathway was activated by ACTN4 downregulation (Figure 8D). To thoroughly understand how ACTN4 silencing induces β-catenin proteasome degradation, we performed the in vitro ubiquitination assay in the presence of E2 ligase enzyme UbcH5a [19]. ACTN4 overexpression caused a loss of poly-ubiquitination accumulation of β-catenin, while its knockdown was found to increase β-catenin ubiquitination and its consequent degradation, presenting as accumulation of ubiquitinated β-catenin. The results indicated that ACTN4 silencing might activate the activity of E2 ligase enzyme UbcH5a, and subsequently accelerate β-catenin proteasome degradation (Figure 8E). Furthermore, the ACTN4-mediated stabilization of β-catenin was tightly correlated with the Akt/GSK-3β signaling. When AKT inhibitor LY294002 was administrated, the decreased β-catenin expression following ACTN4 silencing was aggravated. However, β-catenin reduction induced by ACTN4 silencing was recovered following GSK-3β inhibitor LiCl treatment (Figure 8F). All of these results indicated that ACTN4 sustained breast CSC properties mainly by increasing β-catenin stabilization.

### ACTN4 predicts a poor survival period and basal-like phenotype

**Fig. 9:**
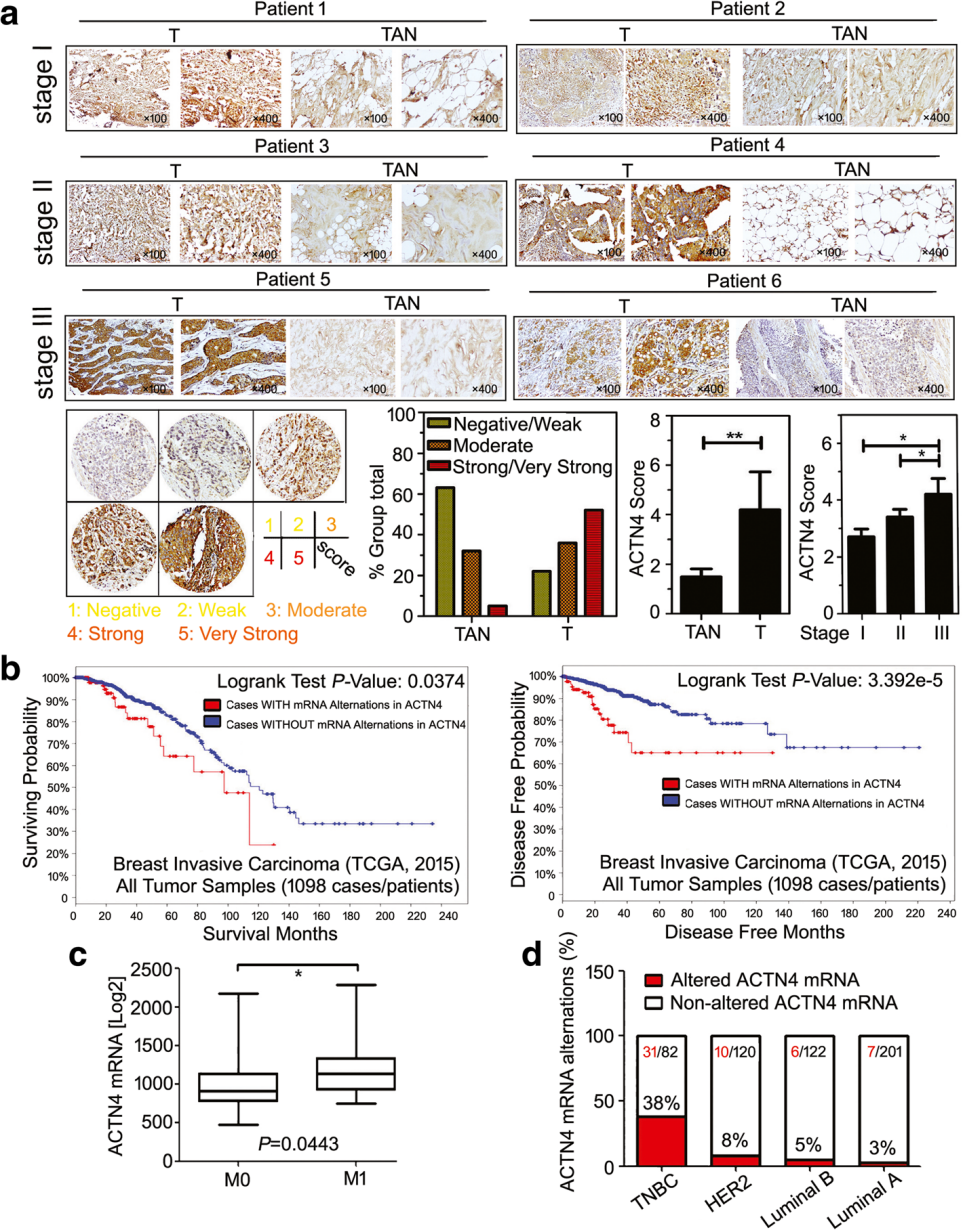
ACTN4 predicts a poor survival and basal-like breast cancer phenotype. **(A)** ACTN4 is overexpressed in breast tumor tissues (T) compared with the tumor adjacent normal (TAN) breast epithelial tissues from the same patient analyzed by the TMA assay, and ACTN4 was significantly correlated with the breast cancer stage; **(B)** Data analysis from the TCGA database implied that ACTN4 mRNA predicted a poor overall survival (OS) **(a)** and disease free survival (DFS) **(b)** in the 1098 breast cancer patients; **(C)** ACTN4 expression was significantly elevated in the metastatic phenotypes (*P* = 0.0443, *P < 0.05) and **(D)** the TNBC patients (38%)

To determine the ACTN4 tissue distribution and expression pattern in human breast tumors, we investigated the expression of the ACTN4 protein in a TMA containing 60 primary breast cancers and paired tumor adjacent normal (TAN) mammary tissues. As shown in representative images, ACTN4 was slightly expressed in the normal mammary tissues, whereas its expression was gradually increased in stages I–III breast cancer (Fig. 9a). These data strongly suggested that ACTN4 expression plays critical roles in breast cancer and might be a potential prognosis biomarker.

Furthermore, 1098 patients from The Cancer Genome Atlas (TCGA) invasive breast carcinoma cancer study (TCGA, 2015) were analyzed to evaluate the oncogenic alterations of ACTN4. The mRNA alterations (amplification, mutation, and/or overexpression) of *ACTN4* occurred in 113 (10%) of the 1098 patients (data not shown). Cases with ACTN4 alterations had a significantly decreased median overall survival (123.3 months vs 97.9 months, *p* = 0.0374). The 3-year and 5-year overall survival of cases with alternated ACTN4 expression was 81.8 months and 64.5 months, respectively. Elevated ACTN4 mRNA expression was correlated with the shorter disease-free survival of patients (*p* = 3.392e-5) **(**Fig. 9b). In addition, comparison between M0 and M1patients demonstrated that cases with metastatic disease had greater ACTN4 mRNA expression (*p* = 0.0443) (Fig. 9c). TNBC phenotypes, which are usually enriched for CD44^+^/CD24^−^ CSCs, also displayed higher ACTN4 expression than other breast cancer subtypes (Fig. 9d). Overall, ACTN4 promotes breast cancer progression and metastasis, and is an independent prognostic marker associated with the poor clinical outcome in breast cancer patients.

## Discussion

DARTS strategy is a novel drug target identification system based on the susceptibility difference to proteolysis between single drug and drug-protein complex [23]. Compared with other affinity-based target identification methods, the key advantage of DARTS is that it does not require ligand modification. Therefore, DARTS is not limited by chemical structure. Here, we applied the DARTS technique to identify ACTN4 as the direct bound protein of EA in breast CD44^+^/CD24^−^ phenotypes. The successful target identification of DARTS strategy is dependent on two factors: the target of the small molecule should be highly abundant in cells, and the identified protein should not be extremely sensitive or resistant to the proteases applied [18]. This indicates that ACTN4 should be a highly abundant protein in breast CSCs, and would be strongly protected by EA from proteolysis and resulted in detectable differences presented as clear variable bands in Fig. 3A. In other words, ACTN4 is one of the most abundant and important targets of EA in breast CSCs, and this is not to exclude the existence of any other possible targets of EA in cancer cells. According to literature reports, EA had inhibition effects on multiple targets of cancer cells, such as VEGFR-2 [14], STAT3 [28], TGF-β [29], and NF-κB [30], etc. However, this is the first study to demonstrate the direct target of EA in cancer cells, and a more comprehensive strategy, such as network pharmacology, might be used to establish the anti-cancer network signaling of EA in the future.

ACTN4, an actin-binding protein, has been described to exist in at least 2 different subcellular locations: the cytosol and nucleus. Shao H et al. proposed ACTN4 was largely responsible for the spreading, motility, and contractility of fibroblasts [31]. Additionally, Honda K et al. demonstrated its potent ability to increase cell motility and promote lymph node metastasis in colorectal cancer [32]. Consistent with their findings, abnormal ACTN4 expression was also correlated to increased tumor invasiveness and metastasis in breast, esophageal, pancreatic, ovarian, and lung carcinomas, indicating that actinin-4 is a promising biomarker for cancer invasion and predictive indicator for patients with metastatic cancer diseases [33–36]. Several studies also identified the crucial role of ACTN4 in transcriptional regulation. ACTN4 transcriptionally potentiated myocyte enhancer factor-2 by antagonizing histone deacetylase [37]. ACTN4 could also serve as a NF-κB co-activator by interacting with its RelA/p65 subunit [38]. More importantly, ACTN4 and E-cadherin might compete for the same binding domain of β-catenin [39]. ACTN4 was found to promote EMT and tumorigenesis by regulating Snail expression and the Akt pathway in cervical cancer [40]. However, few studies have revealed the mechanisms of ACTN4-mediated CSC activities and tumor metastasis in breast cancer.

In the present study, we demonstrated at least 4 lines of evidence supporting the crucial roles of ACTN4 in facilitating CSCs and metastasis in breast cancer. On the threshold, we identified ACTN4 as the direct target of EA in breast CSCs using the DARTS strategy. Further validation suggested that EA administration significantly suppressed ACTN4 expression in vitro and in vivo in the breast cancer model, accompanied by CSC suppressive effects. Next, increased expression of ACTN4 predicted poor differentiation, high metastatic potential, and short overall survival and disease-free survival durations. ACTN4 expression was strongly expressed in the TNBC subtype, which is usually enriched for CD44^+^/CD24^−^ CSCs. Thirdly, CD44^+^/CD24^−^/ACTN4^+^ phenotypes showed a greater ESA^+^ proportion, increased tumorsphere-formation ability, and stronger in vivo tumorigenesis potential in mice when compared with the CD44^+^/CD24^−^/ACTN4^−^ populations. Lastly, ACTN4 sustained breast CSC properties mainly by β-catenin stabilization. Furthermore, Charpentier et al. recently identified that breast stem-like cells display higher levels of microtentacles (McTN), tubulin-based protrusions of the plasma cell membrane that aids cell reattachment [41]. Given that some studies proposed that ACTN4 was preferentially localized in moving structures, such as dorsal ruffles, lamellipodia, and filopodia [39], it is of great significance to further investigate the expression of ACTN4 expression in McTNs.

In addition, our study clearly validated that ACTN4 establishes a direct association between CSC phenotype and metastatic breast cancer. There mainly exists 2 different strategies for CSC isolation and identification [42–44]. The first one is based on unique surface markers in CSCs. Fluorescence-activated cell sorting analysis (FACS) and/or magnetic-activated cell sorting analysis (MACS) are designed to isolate CSCs expressing specific biomarkers, such as CD44^+^, CD24^−^, EpCAM^+^, and ALDH^hi^, etc. [45, 46]. The second strategy is to utilize biofunctions of CSCs. CSC-like cells can be selected by their resistance to chemodrugs, EMT induction, or 3-D sphere culture systems [47, 48]. Nevertheless, functional stem-like cells are not real CSCs and possibly require further purification by additional biomarkers. Our study identified that approximately 100% of the basal-like phenotype MDA-MB-231 cells are CD44^+^/CD24^−^ subpopulations, among which CD44^+^/CD24^−^/ACTN4^+^ cells exhibited better CSC-like properties (high self-renewal properties, adherence-promoting capacity, and tumorigenic potential with very few cells) compared with the unfractioned parts. This finding was consistent with recent studies that showed the presence of CD44^+^/CD24^−^ cells does not correlate with tumorigenicity [49], distant metastasis or clinical outcome [26]. Thus, it is reasonable that combined utilization of ACTN4 and CD44^+^/CD24^−^ markers might be more sufficient to isolate metastatic cells with CSC properties in breast cancer.

## Conclusion

In conclusion, our study not only identified ACTN4 as the direct target of EA, but it also highlighted its significant role in mediating breast cancer metastasis and CSCs’ properties. Mechanism exploration further revealed that interruption of ACTN4/β-catenin interaction will result in the activation of β-catenin proteasome degradation. It is promising to develop ACTN4 targeting strategies to predict or inhibit breast cancer metastasis in the future, especially for the basal-like phenotypes.

## Additional files

Additional file 1: Table S1. Primers for Real-time PCR analysis. **Table S2.** Tumorigenic ability of CD44^+^/CD24^−^/ACTN4^+^ cells (DOC 51 kb)

Additional file 2: Figure S1. (A) Cancer progression was slowed down if the EA treatment was started after tumor growth in MMTV-PyMT mice at ages ranging from 6th to 15th weeks (*P < 0.05, values represented as the Mean ± SD, *n* = 3);** (B)** EA treatment did not cause a significant bodyweight loss compared to the vehicle group. (JPEG 241 kb)

Additional file 3: Figure S2. Flow cytometry detection indicated that EA dose-dependently arrested cell cycle mainly at the S&G2/M phases, and induced apoptosis in breast cancer cells MDA-MB-231, BT-549 and MCF-7. (JPEG 703 kb)

Additional file 4: Figure S3 and S4. The wound healing and chamber invasive assay revealed that breast cancer cell migration and invasion were inhibited by EA in a time- and dose-dependent manner. (ZIP 2039 kb)

Additional file 5: Figure S5. RNA-seq showed that EA treatment and ACTN4 knockdown exhibited similar changes of target genes by BGISEQ-500 analysis. **(A)** Heat map columns describing the hierarchical clustering of EA-treated or ACTN4 knockdown group compared to control, respectively (log 2 fold change ≥ 1.2, *P* ≤ 0.05); **(B)** Venn diagrams of up-regulated and down-regulated DEGs beween EA treatment and ACTN4 knockdown groups; **(C)** GO terms analysis of the indicated DEGs containing 3 aspects including molecular function, cellular component and biological process; **(D)** KEGG pathway enrichment analysis of the indicated DEGs. (JPEG 5607 kb)

## Abbreviations

ALDH: Aldehyde dehydrogenase
BC: breast cancer
BSA: Bovine serum albumin
CHIP: Chromatin immunoprecipitation
CHX: Cycloheximide
CSCs: Breast cancer stem cells
DAPI: 4′,6-diamidino-2-phenylindole
DARTS: Drug affinity responsive target stability
DEAB: Diethylaminobenzaldehyde
DMSO: Dimethylsulfoxide
EA: Ellagic acid
ELDA: Extreme limiting dilution analysis
EMT: Epithelial-mesenchymal transition
FACS: Fluorescence-activated cell sorting analysis
H&E: Hematoxylin and eosin
LiCl: Lithium chloride
MACS: Magnetic-activated cell sorting analysis
MTT: 3-(4, 5-dimethylthiazol −2-yl) -2, 5-diphenyltetrazolium bromide
TAN: Tumor adjacent normal
TCGA: The cancer genome atlas
TMA: Tissue microarray
