# Correction to: Direct inhibition of ACTN4 by ellagic acid limits breast cancer metastasis via regulation of β-catenin stabilization in cancer stem cells

**DOI:** 10.1186/s13046-022-02341-1

**Published:** 2022-03-31

**Authors:** Neng Wang, Qi Wang, Hailin Tang, Fengxue Zhang, Yifeng Zheng, Shengqi Wang, Jin Zhang, Zhiyu Wang, Xiaoming Xie

**Affiliations:** 1 Department of Breast Oncology, Sun Yat-sen University Cancer Center; State Key Laboratory of Oncology in South China; Collaborative Innovation Center of Cancer Medicine, Guangzhou, People’s Republic of China; 2 Guangdong Provincial Hospital of Chinese Medicine, The Second Clinical Medical College & The Research Center of Integrative Medicine, Guangzhou University of Chinese Medicine, Guangzhou Shi, China

**Correction to: J Exp Clin Cancer Res 36, 172 (2017)**

**https://doi.org/10.1186/s13046-017-0635-9**

Following publication of the original article [1], minor errors we identified in Fig. 6d and Fig. S4; specifically:

- Figure 6d: incorrect image used for shCtrl scratch image (24 h); correct image now used
- Fig. S4: incorrect images used for the invasive MDA-MB-231 cells (25 μM EA); correct images now used

**Fig. 6:**
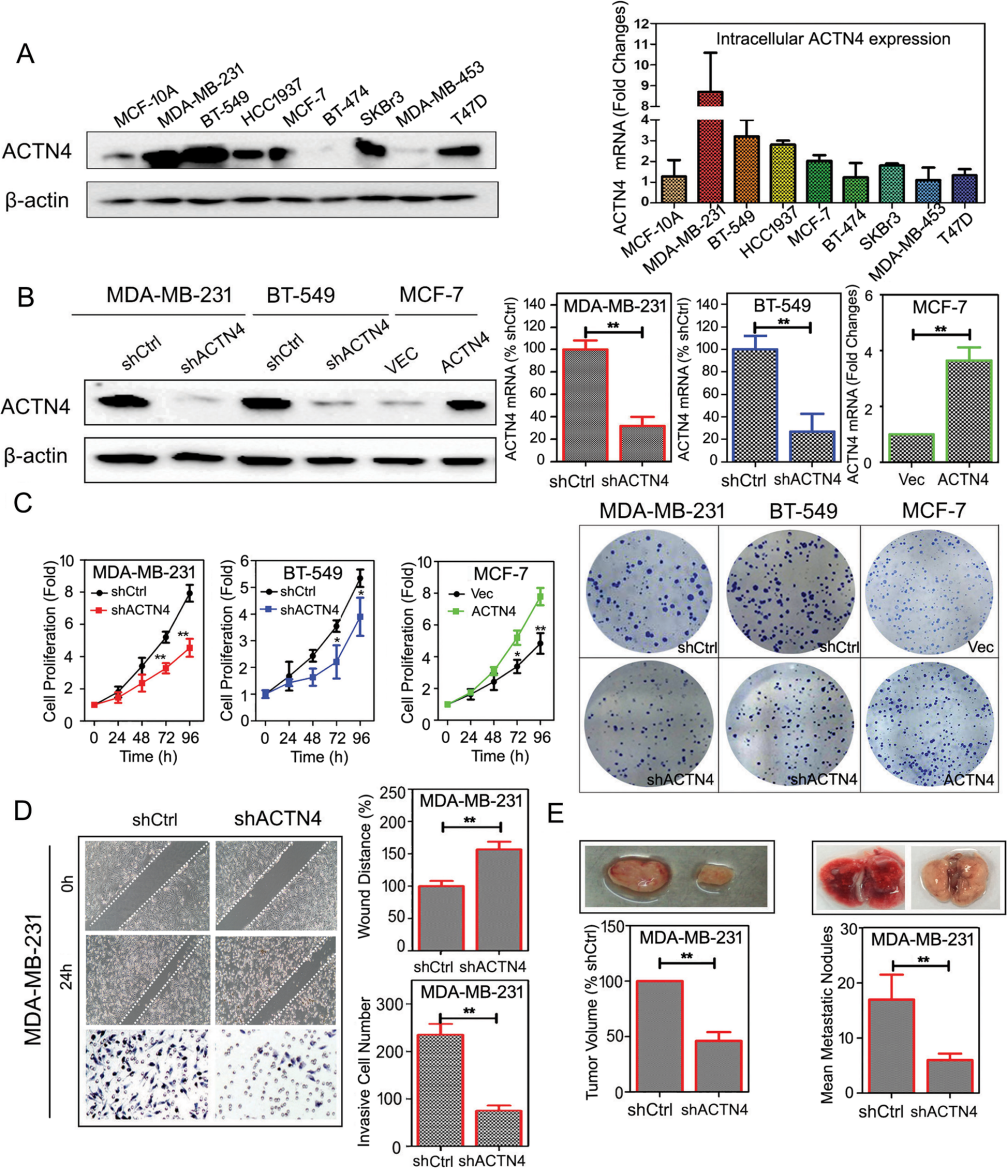
ACTN4 promotes breast cancer proliferation and metastasis *in vitro* and *in vivo*. **a** Intracellular expression of ACTN4 was determined by Western blot (left) and real-time PCR (right) analysis, respectively; **b** ACTN4 expression was modified by transfecting recombinant plasmid or its shRNA in breast cancer cells and subjected to Western blotting (left) and real-time PCR (right) validation; **c** MTT and colony formation assay showed that ACTN4 silencing abrogated breast cancer cell proliferation while its overexpression promoted cell growth; **d** ACTN4 silencing inhibited the migration and invasion abilities of MDA-MB-231 cells; **e** ACTN4 silencing inhibited breast cancer growth and lung metastasis *in vivo* (*P < 0.05, **P < 0.01 versus control, values represented as the mean ± SD, n = 3)

The corrected figures, produced using the original data, are given here. The correction does not have any effect on the final conclusions of the paper.

## Supplementary Information

**Additional file 4.** The wound healing and chamber invasive assay revealed that breast cancer cell migration and invasion were inhibited by EA in a time- and dose-dependent manner.
