# Supplementary material for: Direct inhibition of ACTN4 by ellagic acid limits breast cancer metastasis via regulation of β-catenin stabilization in cancer stem cells
Source: J Exp Clin Cancer Res. 2017 Dec 2;36:172. doi: 10.1186/s13046-017-0635-9 (PMC5712102; doi:10.1186/s13046-017-0635-9)
Supplement: Supplementary file 1 — Primers for Real-time PCR analysis. Table S2. Tumorigenic ability of CD44+/CD24−/ACTN4+ cells (DOC 51 kb) [file 13046_2017_635_MOESM1_ESM.doc]

| **Supplementary Table 1: Primers for Real-time PCR analysis** | |
| --- | --- |
| **β-catenin** | (F): 5’-GCTTTCAGTTGAGCTGACCA-3’ |
|  | (R): 5’-CAAGTCCA AGATCAGCAGTCTC-3’ |
| **Nanog** | (F): 5’-ATGCCTCACACGGAGACTGT-3’ |
|  | (R): 5’-CAGGGCTGTCCTGAATAAGC-3’ |
| **c-Myc** | (F): 5’-GCTGCTTAGACGCTGGATTT-3’ |
|  | (R): 5’-TAACGTTGAGGGGCATCG-3’ |
| **Oct-4** | (F): 5’-CAATTTGCCAAGCTCCTGA-3’ |
|  | (R): 5’-AGATGGTCGTTTGGCTGAAT-3’ |
| **survivin** | (F): 5’-CAATTTGCCAAGCTCT GA-3’ |
|  | (R): 5’-AGATGGTCGTTTGGCTGAAT-3’ |
| **CyclinD1** | (F): 5’-AGGCCGGTGCTGAGTATGTC-3’ |
|  | (R): 5’-TGCCTGCTTCACCACCTTCT-3’ |
| **ZEB1** | (F): 5’- TTCAAACCCATAGTGGTTGCT -3’ |
|  | (R): 5’- TGGGAGATACCAAACCAACTG -3’ |
| **ZEB2** | (F): 5’- AACAACGAGATTCTACAAGCCTC -3’ |
|  | (R): 5’- TCGCGTTCCTCCAGTTTTCTT -3’ |
| **Snail1** | (F): 5’-ACCACTATGCCGCGCTCTT-3’ |
|  | (R): 5’- GGTCGTAGGGCTGCTGGAA-3’ |
| **Slug** | (F): 5’- TGTTGCAGTGAGGGCAAGAA -3’ |
|  | (R): 5’- GACCCTGGTTGCTTCAAGGA -3’ |
| **ACTN4** | (F): 5’- GAACGACCGGCAGGGTGAGG-3’ |
|  | (R): 5’- TCGGTGGTCTCCCGCGACAT-3’ |
| **Twist1** | (F): 5’- TCTACCAGGTCCTCCAGAGC -3’ |
|  | (R): 5’- CTCCATCCTCCAGACCGAGA -3’ |
| **Twist2** | (F): 5’- GCAAGAAGTCGAGCGAAGAT -3’ |
|  | (R): 5’- GCTCTGCAGCTCCTCGAA -3’ |
| **β-actin** | (F): 5’-CCAACCGCGAGAAGATGA-3’ |
|  | (R): 5’-CCAGAGGCGTACAG GGATAG-3’ |

**Supplementary Table 2: Tumorigenic ability of CD44^+^/CD24^-^/ACTN4^+^ cells**

| CD44^+^CD24^-^ | No. tumors/No. injection | | | | | |
| --- | --- | --- | --- | --- | --- | --- |
|  | No. cells per injection | | | Tumor initiating cell frequency | | |
|  | 100000 | 10000 | 1000 | Estimate | 95% CI | *P* Value |
| ACTN4^+^ | 6/6 | 6/8 | 6/10 | 1/3738 | (1/1740-1/8030) | 0.000898 |
| ACTN4^-^ | 5/6 | 5/8 | 4/10 | 1/17655 | (1/7072-1/44077) |  |
