# Supplementary figures and images for: Direct inhibition of ACTN4 by ellagic acid limits breast cancer metastasis via regulation of β-catenin stabilization in cancer stem cells
Source: J Exp Clin Cancer Res. 2017 Dec 2;36:172. doi: 10.1186/s13046-017-0635-9 (PMC5712102; doi:10.1186/s13046-017-0635-9)

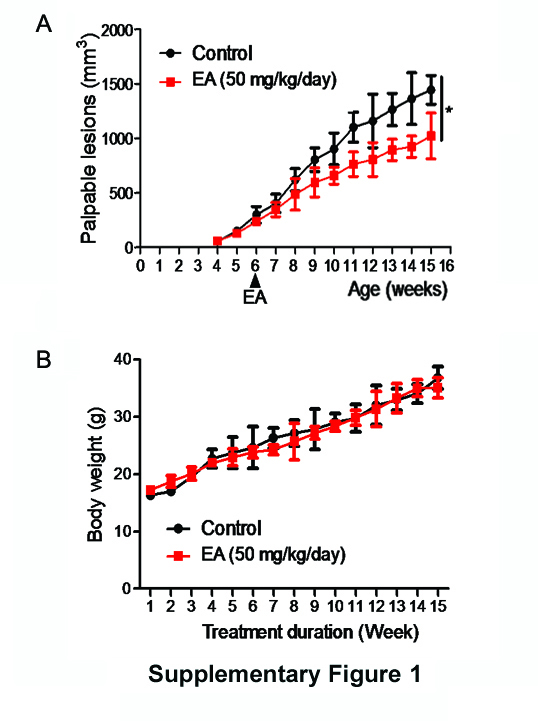

Supplement: Supplementary file 2 — Cancer progression was slowed down if the EA treatment was started after tumor growth in MMTV-PyMT mice at ages ranging from 6th to 15th weeks (*P < 0.05, values represented as the Mean ± SD, n = 3); (B) EA treatment did not cause a significant bodyweight loss compared to the vehicle group. (JPEG 241 kb) [file 13046_2017_635_MOESM2_ESM.jpg]

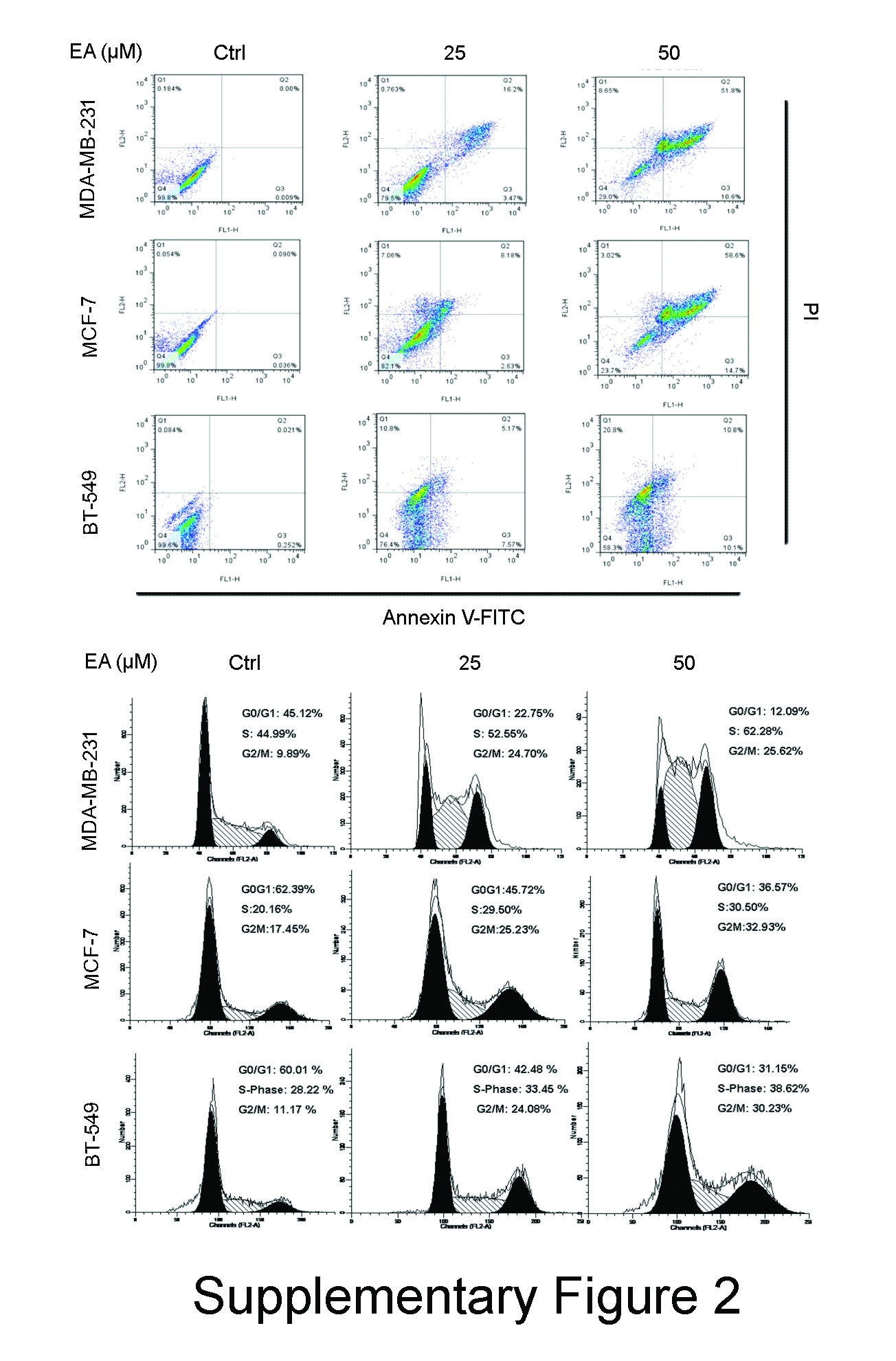

Supplement: Supplementary file 3 — Flow cytometry detection indicated that EA dose-dependently arrested cell cycle mainly at the S&G2/M phases, and induced apoptosis in breast cancer cells MDA-MB-231, BT-549 and MCF-7. (JPEG 703 kb) [file 13046_2017_635_MOESM3_ESM.jpg]

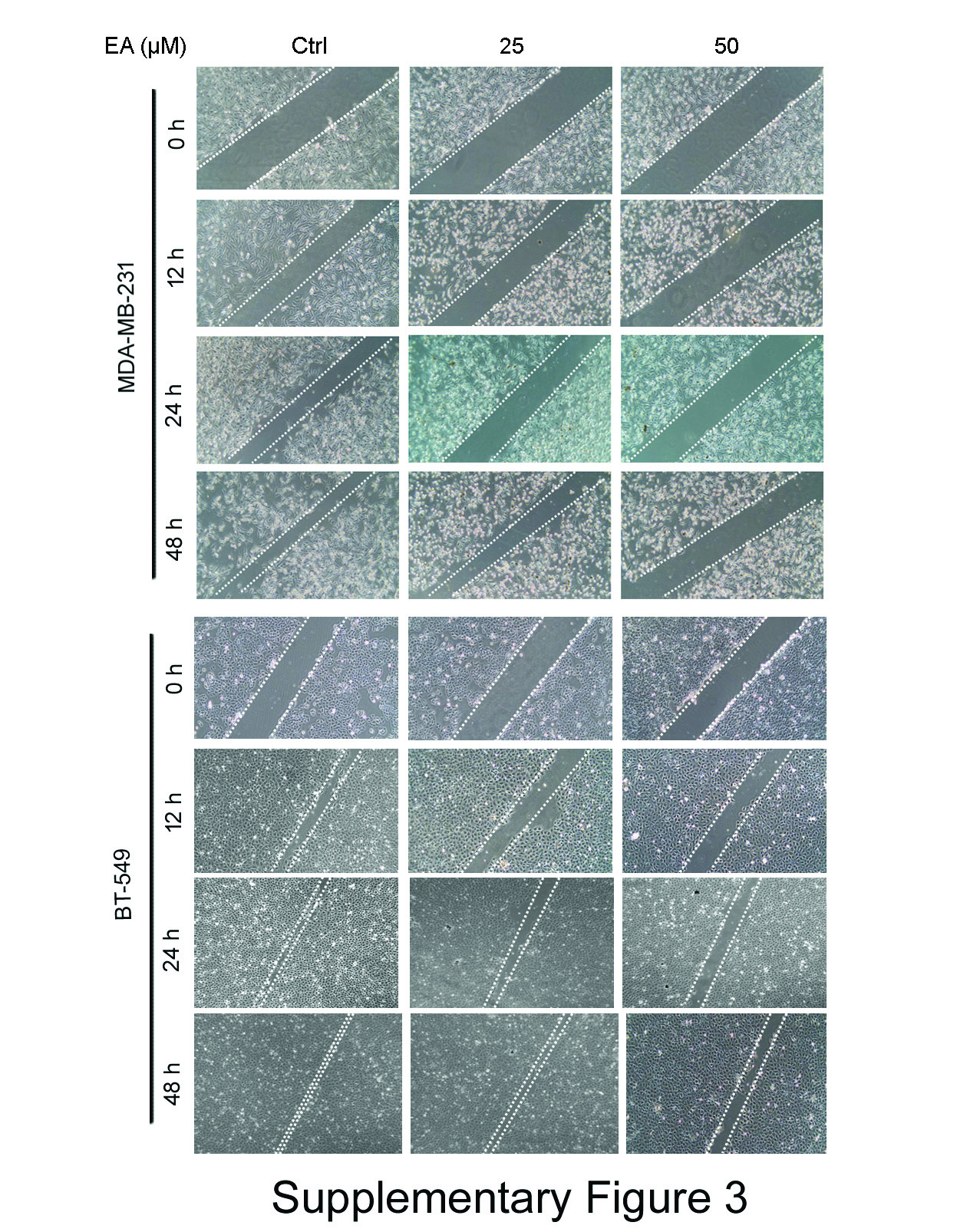

Supplement: Supplementary file 4 — The wound healing and chamber invasive assay revealed that breast cancer cell migration and invasion were inhibited by EA in a time- and dose-dependent manner. (ZIP 2039 kb) [file 13046_2017_635_MOESM4_ESM.zip › Sfigure 3.jpg]

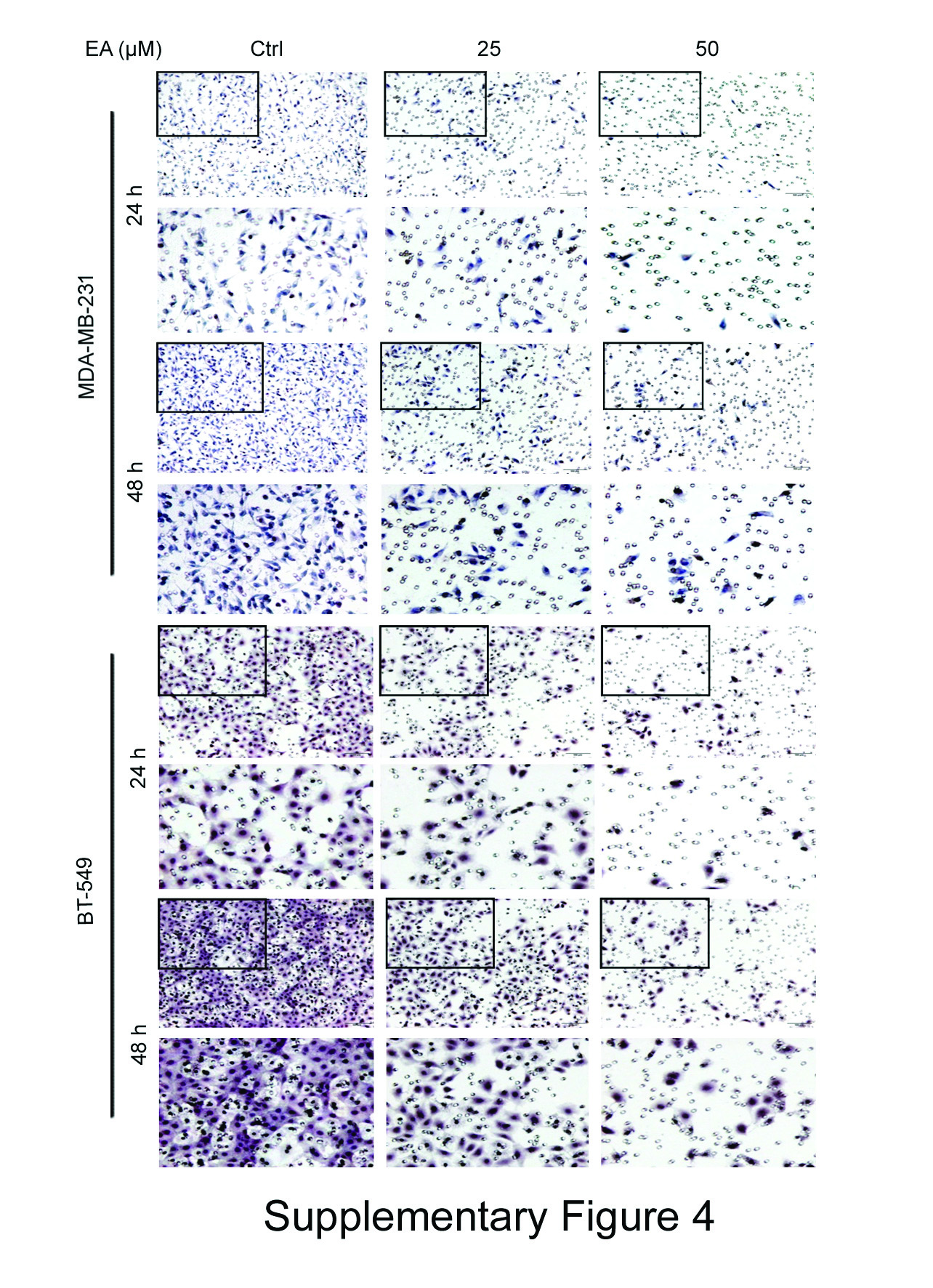

Supplement: Supplementary file 4 — The wound healing and chamber invasive assay revealed that breast cancer cell migration and invasion were inhibited by EA in a time- and dose-dependent manner. (ZIP 2039 kb) [file 13046_2017_635_MOESM4_ESM.zip › Sfigure 4.jpg]

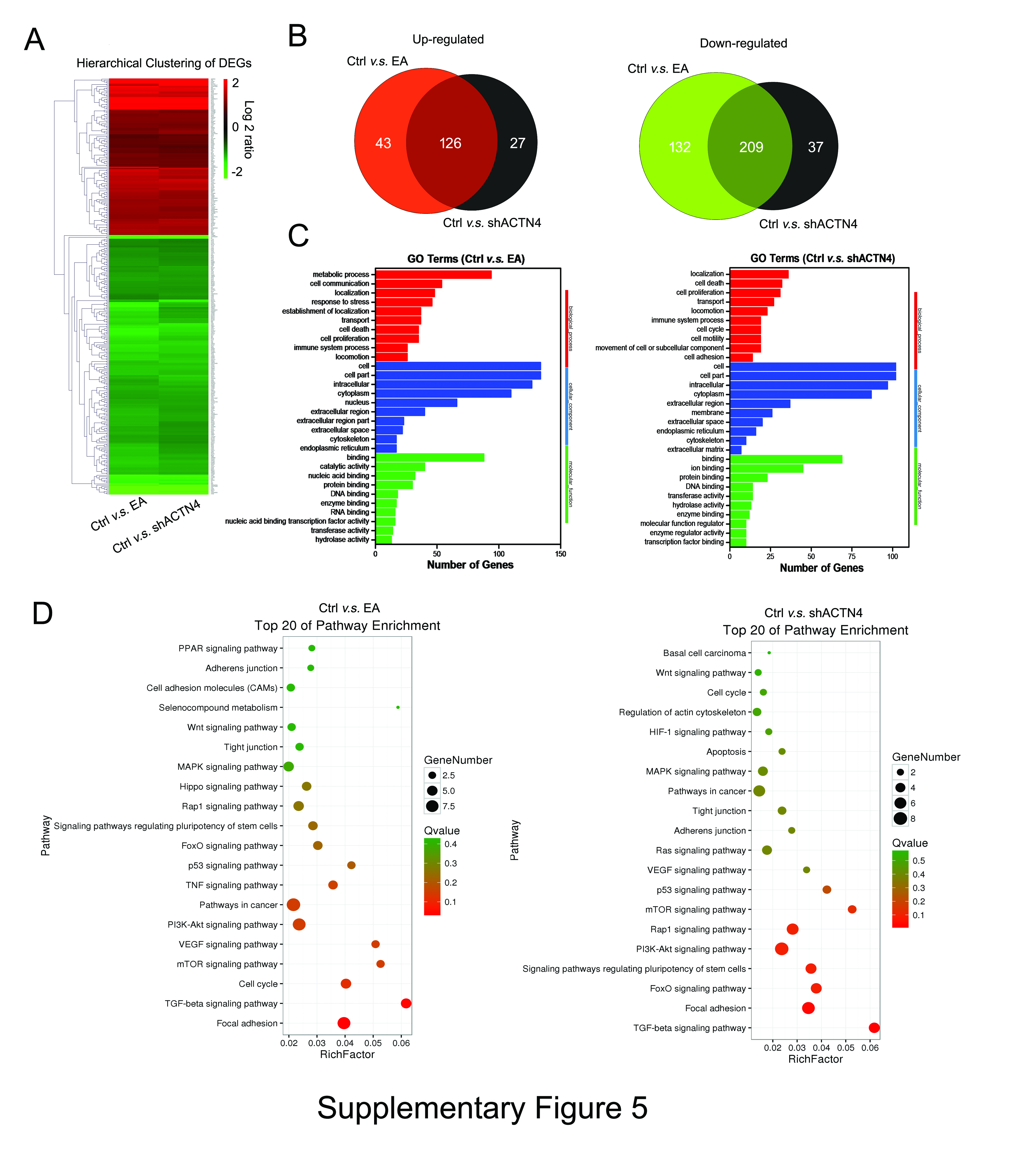

Supplement: Supplementary file 5 — RNA-seq showed that EA treatment and ACTN4 knockdown exhibited similar changes of target genes by BGISEQ-500 analysis. (A) Heat map columns describing the hierarchical clustering of EA-treated or ACTN4 knockdown group compared to control, respectively (log 2 fold change ≥ 1.2, P ≤ 0.05); (B) Venn diagrams of up-regulated and down-regulated DEGs beween EA treatment and ACTN4 knockdown groups; (C) GO terms analysis of the indicated DEGs containing 3 aspects including molecular function, cellular component and biological process; (D) KEGG pathway enrichment analysis of the indicated DEGs. (JPEG 5607 kb) [file 13046_2017_635_MOESM5_ESM.jpg]
