# Supplementary figures and images for: Correction to: Direct inhibition of ACTN4 by ellagic acid limits breast cancer metastasis via regulation of β-catenin stabilization in cancer stem cells
Source: J Exp Clin Cancer Res. 2022 Mar 31;41:118. doi: 10.1186/s13046-022-02341-1 (PMC8969376; doi:10.1186/s13046-022-02341-1)

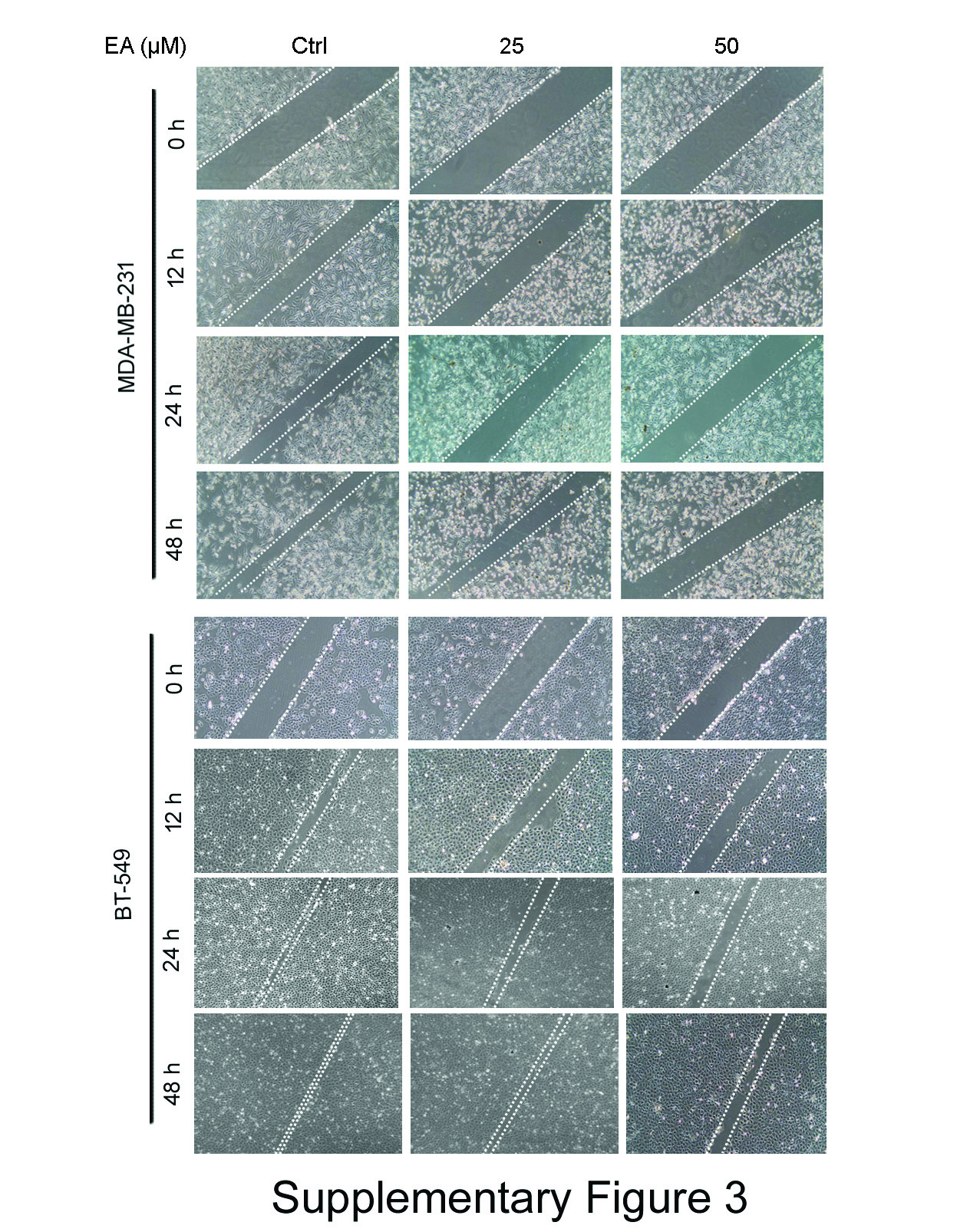

Supplement: Supplementary file 1 — Additional file 4. The wound healing and chamber invasive assay revealed that breast cancer cell migration and invasion were inhibited by EA in a time- and dose-dependent manner. [file 13046_2022_2341_MOESM1_ESM.zip › 13046_2017_635_MOESM4_ESM/Sfigure 3.jpg]

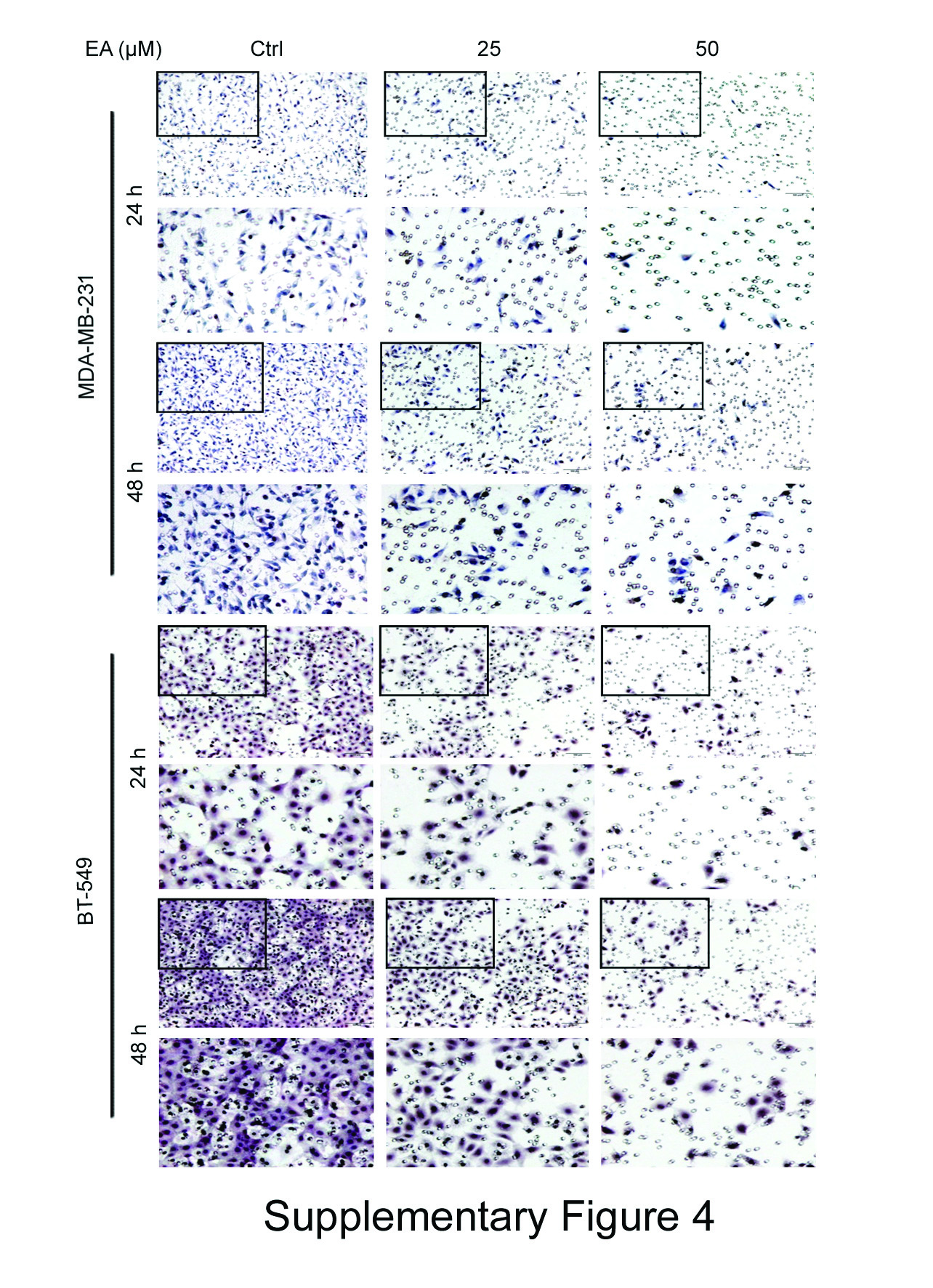

Supplement: Supplementary file 1 — Additional file 4. The wound healing and chamber invasive assay revealed that breast cancer cell migration and invasion were inhibited by EA in a time- and dose-dependent manner. [file 13046_2022_2341_MOESM1_ESM.zip › 13046_2017_635_MOESM4_ESM/Sfigure 4.jpg]
